# Locus-specific expression of transposable elements in single cells with CELLO-seq

**DOI:** 10.1038/s41587-021-01093-1

**Published:** 2021-11-15

**Authors:** Rebecca V Berrens, Andrian Yang, Christopher E Laumer, Aaron TL Lun, Florian Bieberich, Cheuk-Ting Law, Guocheng Lan, Maria Imaz, Joseph S Bowness, Neil Brockdorff, Daniel Gaffney, John C Marioni

**Affiliations:** 1 Cancer Research UK Cambridge Institute, University of Cambridge, Li Ka Shing Centre, Robinson Way, Cambridge CB2 0RE, UK; 2 European Molecular Biology Laboratory, European Bioinformatics Institute, Wellcome Genome Campus, Hinxton CB10 1SD, Cambridge, UK; 3 Wellcome Sanger Institute, Wellcome Genome Campus, Hinxton CB10 1SA, UK; 4 Developmental Epigenetics, Department of Biochemistry, University of Oxford, South Parks Road, Oxford OX1 3QU, UK

## Abstract

Transposable Elements (TEs) regulate diverse biological processes, from early development to cancer. Expression of young TEs is difficult to measure with next generation single-cell sequencing technologies as their highly repetitive nature means that short cDNA reads cannot be unambiguously mapped to a specific locus. CELLO-seq combines long-read scRNA sequencing with computational analyses to measure TE expression at unique loci. We use CELLO-seq to assess the widespread expression of TEs in 2-cell mouse blastomeres as well as human induced pluripotent stem cells (hiPSCs). Across both species, old and young TEs showed evidence of locus-specific expression, with simulations demonstrating that only a small number of very young elements in the mouse could not be mapped back to the reference with high confidence. Exploring the relationship between the expression of individual elements and putative regulators revealed large heterogeneity, with TEs within a class showing different patterns of correlation, suggesting distinct regulatory mechanisms.

## Introduction

Understanding the complete picture of gene expression in single cells is necessary to characterize distinct cellular states in normal development and disease^1^. Single-cell RNA-sequencing (scRNA-seq) has recently emerged as a tool for characterising the transcriptomes of individual cells, facilitating projects such as the Human Cell Atlas ^2,3^ However, the majority of scRNA-seq protocols use short-read sequencing and, moreover, are biased towards tagging either the 3’ or 5’ end of a transcript. Consequently, the ability to profile individual isoforms, to assay allele specific expression and to study the expression of individual young transposable elements in single-cells has generally remained challenging ^4–12^. Moreover, although several approaches have been used in bulk RNA-seq to study young transposable element expression specifically^13–15^, these approaches have not been applied to single cells and concentrate on targeted enrichment of L1s, therefore losing the possibility to study expression of putative regulators of TEs and transcription of young TEs in the same cell.

The expression of TEs is being increasingly recognised as playing a key role in multiple biological processes, with a recent focus being their function in early mammalian development ^16^. More generally, half of the mammalian genome is estimated to consist of repetitive DNA elements, with TEs contributing to 45% of the human and 37.5% of the mouse genome ^17,18^. TEs can be classified based upon their method of transposition, with Class 1 elements (including long and short interspersed elements (LINEs and SINEs) and Long Terminal Repeats (LTRs)) that transpose via RNA intermediates and Class 2 elements, or DNA transposons, that transpose via a DNA intermediate through a so-called cut- and-paste mechanism ^3^. Most TEs are silenced, existing as fragmented copies in the genome. However, 1-2% of TEs are young, full-length and mobile in both the mouse (e.g. SINE-B1, L1MdA, L1MdF, MERVL and IAPs elements) and the human (e.g., L1HS, SVA-E, AluYb8 and HERV-int) genome ^19–23^. Epigenetic modifications repress TEs in most somatic cells but they can be transcribed during early development and global epigenetic reprogramming ^24,25^. Recent work on LINE expression during early development has hinted at a role for retrotransposons in regulating gene expression via an unknown mechanism ^26,27^. However, due to the highly repetitive nature of TEs across the genome, short cDNA reads cannot be unambiguously mapped to a specific young TE locus-of-origin. Therefore, measures of young TE expression have been aggregated across multiple elements at a family or subfamily level, limiting the ability to understand the mechanisms by which they might regulate the expression of messenger RNAs and hence normal development at single cell level.

**Figure 1:**
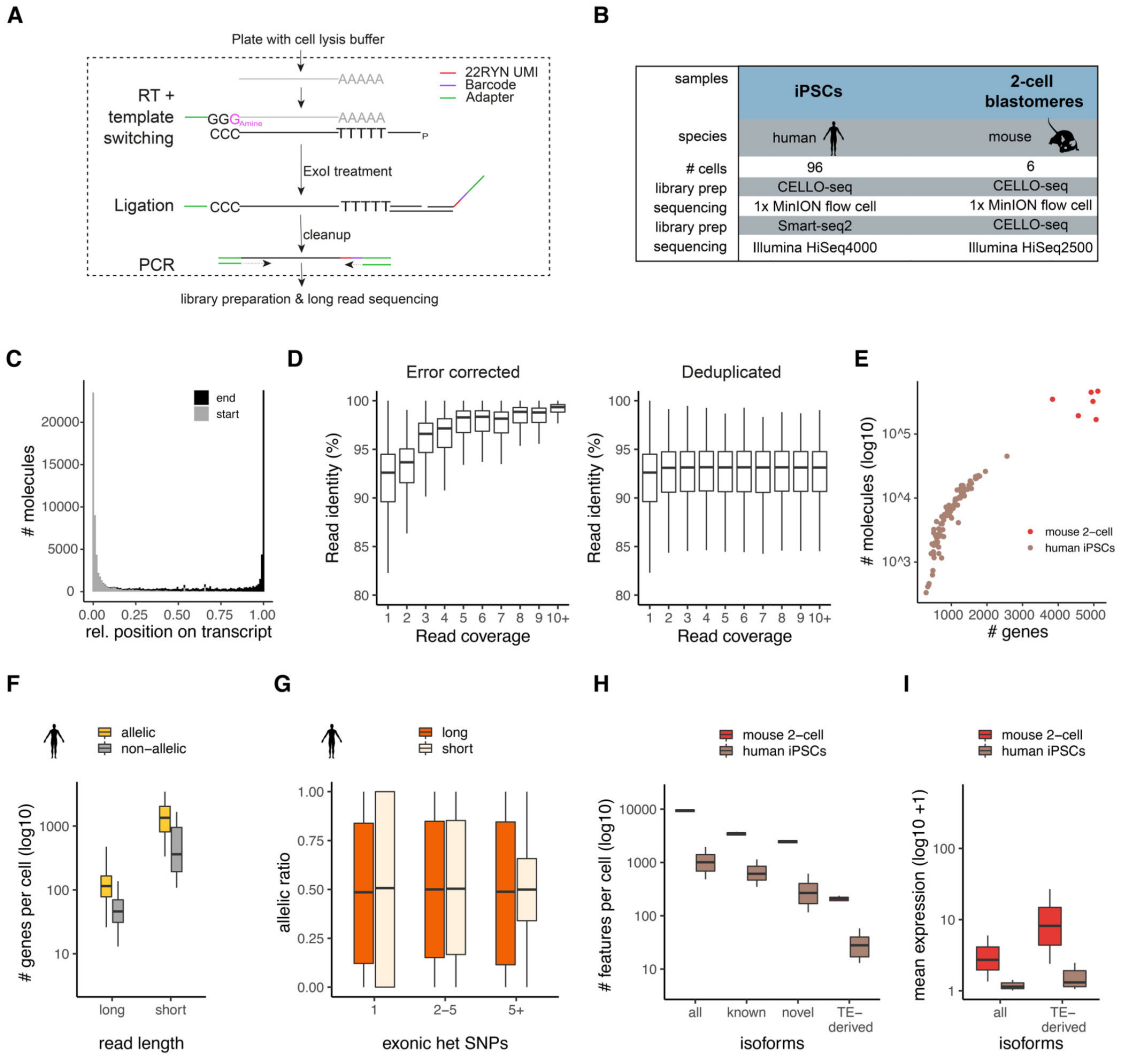
CELLO-seq overview and ability to study allelic and isoform expression. (A) CELLO-seq protocol. Single-cells are sorted into plates and lysed. Poly-A mRNA is reverse transcribed with a template switch oligo. Following exonuclease treatment, splint oligos with 22-RYN UMIs and cellular barcodes are ligated onto first strand cDNA. Before PCR libraries are cleaned up. (B) datasets generated in this study. (C) transcript coverage over reads with relative start and end position for each transcript averaged over hiPSCs (n=96 cells). (D) Boxplots of read identity (y-axis) by read coverage (x-axis) with error corrected (left) versus deduplicated (right) data. The Y-axis is adjusted to cover 80-100% of read identity and outliers are removed in boxplot. Error corrected coverage 1x (n=1506129), 2x (n=174058), 3x (n=83999), 4x (n=42874), 5x (n=24950), 6x (n=16228), 7x (n=11195), 8x (n=8297), 9x (n=6228), 10+x (n=58361), deduplicated coverage 1x (n=1506287), 2x (n=174964), 3x (n=84137), 4x (n=43088), 5x (n=24982), 6x (n=16275), 7x (n=11247), 8x (n=8309), 9x (n=6247), 10+x (n=58420). (E) Scatter plot of number of genes per error corrected UMI sequence for mouse (n=6) and human (n=96). (F) boxplot of genes per cell (y-axis) with CELLO-seq or Smart- seq2 of hiPSCs (n=96), split into genes with or without at least one heterozygous genic SNP . (G) boxplot of allelic ratio = allele1/(allele1+allele2) (y-axis) by exonic heterozygous SNPs with CELLO-seq and Smart-seq2 of hiPSCs. Short reads: 1 SNP (n=6587), 2-5 SNPs (n=10023), >5 SNPs (n=8088), Long reads: 1 SNP (n=1072), 2-5 SNPS (n=1796), >5 SNPs (n=1710). (H) boxplot of number of isoforms per cell (y-axis) in mouse blastomeres or hiPSCs. Isoforms were grouped in either all, (known) when overlapping ENSEMBL transcript-ID, (novel), when not overlapping with an ENSEMBL transcript-ID and (TE-derived) when isoforms overlapped repeat and ENSEMBL transcript-ID, or if isoforms overlapped with repeats but did not overlap with genic exons. Human (n=96 cells), mouse (n=6 cells). (I) boxplot of mean expression of isoforms (y-axis) by classification of (H) in mouse blastomeres or hiPSCs. Mean expression level was calculated from logcounts of all cells. The boxplots shown in D,F-I show the median, first and third quartiles as a box, and the whiskers indicate the most extreme data point within 1.5 lengths of the box.

After confirming that we could not map young L1s at unique loci with current long read single cell RNAseq methods based on 10X and ONT sequencing, we developed an experimental protocol for single CELl LOng read RNA sequencing (CELLO-seq) (Fig 1A), and an associated computational framework, which combines the benefits of existing scRNA-seq protocols with advances in long-read sequencing as implemented by Oxford Nanopore Technologies (ONT) and Pacific Biosciences (PacBio). Our approach uses long Unique Molecular Identifiers (UMIs) to counteract the high error rate associated with ONT reads relative to Illumina sequencing. By profiling mouse blastomeres from 2-cell stage embryos and a larger set of human Induced Pluripotent Stem Cells (hiPSCs) CELLO-Seq uncovers unexpected heterogeneity in expression of individual elements within specific repeat classes. Moreover, these differences are associated with different upstream epigenetic regulators, suggesting different modes of regulation.

## Results

### CELLO-seq produces full-length molecule level cDNAs

To investigate whether existing protocols allow the expression of young transposable elements could be measured at unique loci in single cells, we generated long read single cell RNAseq libraries of mouse embryonic stem cells (mESCs) cultured in 2i conditions with ScNaUMI-seq^28^. Previous studies have demonstrated that families of young Transposable Elements (TEs) are transcriptionally active at this stage of development ^29,30^. In the ScNaUMI-seq protocol, the error correction of long reads depends upon correctly mapped short reads – consequently, we explored the number of short reads mapped to young TEs. However, all young TEs demonstrated extremely low levels of expression (no or a single read in the majority of cells), indicating that we are not able to reliably measure the expression of individual LINE elements using this or similar approaches (Extended Data Fig S1A). We reasoned that the low level of expression observed was driven by an inability to uniquely map short reads to individual young TEs. Moreover, the ability to directly use long-read sequencing approaches is inhibited by the substantial degree of sequencing error, making it difficult to uniquely map individual reads to specific young TEs.

Motivated by these observations, we developed CELLO-seq, a method that adds a long (22nt) Unique Molecular Identifier (UMI) onto every full-length transcript before profiling expression using only long read sequencing (Fig 1A). The long UMI allows error correction of individual reads, thereby enabling their unique mapping. In brief, we used a 3’-amino blocked template switching oligonucleotide (TSO) to prevent the synthesis of TSO-primed inserts. We also performed the reverse transcription (RT) at high temperature whilst adding RT enhancers to increase cDNA output and the length of transcripts. After RT, we splint-ligated an oligonucleotide containing both a 22-base unique molecular identifier (UMI), and a cellular barcode, to label unique mRNAs. By designing UMIs with a repetitive pattern of RYN (where R and Y represent purine and pyrimidine, respectively), we prevent the presence of homopolymers, which remain inaccurately basecalled in ONT sequencing, although this challenge is receiving continued attention^31^. These long UMIs allow the identification of PCR duplicates, which can be used for deduplication and error correction by producing consensus sequences for each full-length transcript ^28,32^ (Fig 1A). Additionally, and unlike other protocols, we aim for high PCR duplicate numbers, which limits library diversity ^33^ as we require a high number of PCR cycles (Extended Data Fig S1C). These two steps enable us to error-correct transcriptomic libraries sequenced on ONT instruments thus facilitating the generation of high-quality long-reads that can be used for isoform, allelic gene expression analysis and, critically, unlike existing protocols (Extended Data Fig S1A,B) for assaying the expression of young TEs.

To assess the performance of CELLO-seq, we generated two independent datasets, the first comprising six manually isolated, 2-cell stage mouse blastomeres which were deeply sequenced, and the second profiling 96 hiPSCs using shallower sequencing (Fig 1B, Extended Data Fig S1D). Confirming the efficacy of our protocol, in CELLO-seq libraries the majority of reads (75%) were flanked with both TSO and oligo-dT-derived sequences (Extended Data Fig S1E). This contrasts with other recently-published single cell long read protocols, which generated libraries with the 10x protocol before sequencing using ONT or PacBio, where only 40% of reads contained both cellular barcodes and UMIs ^34,35^. Additionally, CELLO-seq produced mostly full-length transcript sequences with the mean read length of mapped reads being 2-2.5kb (Fig 1C, Extended Data Fig S1F).

Subsequently, we processed the CELLO-seq libraries using a computational pipeline, sarlacc, which demultiplexes cell barcodes, trims adapters, identifies UMIs, and error corrects or deduplicates each cDNA molecule (Extended Data Fig S1H, https://github.com/MarioniLab/sarlacc). With sarlacc we were able to error correct the cDNA molecules to obtain reads with higher sequence identity in comparison to deduplication (Fig 1D,Table S1). We measured expression for an average of 4,747 genes (324,991 molecules) per cell for the mouse blastomeres and 960 genes (7,677 molecules) per cell for the hiPSCs, reflecting the different sequencing depths of these experiments (Fig 1E). Comparing our ONT data with Illumina short read sequencing of the same cDNA libraries, we noted that although the depth of sequencing of the CELLO-seq data is lower than data obtained using short read sequencing (Extended Data Fig S1G), we observed a high correlation between the expression of protein-coding genes between short and long read data, irrespective of the gene’s length (Extended Data Fig S1I,J). Additionally, we observed a strong positive correlation between ERCC spike-in concentration and measured read counts in both the long-read (R=0.88) and the short read (R=0.87) data, demonstrating the ability of CELLO-seq to quantitatively measure gene expression levels (Extended Data Fig S1K,L).

To further demonstrate the utility of CELLO-seq, we examined its ability to quantify allele-specific expression by preparing CELLO-seq and Smart-seq2 libraries ^36^ using cells from two human iPSC lines. Using previously generated phased exome-seq data from the same cell lines ^37^ we were able to measure allelic expression for genes with heterozygous SNPs present in coding regions (Fig 1F). As expected, the degree of allele-specific expression (ASE) did not change as a function of the number of exonic SNPs when using the CELLO-seq protocol. By contrast, in the Smart-seq2 data the variability diminishes as a function of the number of SNPs, with more extreme ASE being observed for genes containing only a single exonic SNP (Fig 1G). Consequently, CELLO-seq provides a less biased approach for measuring allele-specific expression than short read strategies.

Next, we probed the ability of CELLO-seq to measure the expression of isoforms in single cells. After processing the error corrected reads with FLAIR ^38^, we were able to measure the expression of ~10,000 and ~1,000 distinct isoforms across the mouse blastomeres and human iPSCs respectively, amongst which ~5,000 and ~300 were novel (Fig 1H). Amongst these, CELLO-seq enabled detection of ~200 and ~20 TE-derived isoforms per cell in mouse blastomeres and hiPSCs, respectively (Fig 1H). The expression of TE-derived isoforms in both datasets was higher than all isoforms (Fig 1I), which warrants further investigation. Hence, not only can CELLO-seq detect novel isoforms in single cells but it can also enable study of the role of TE-derived isoforms at single cell resolution.

### Analysis of TE-derived isoforms and TEs at unique loci

**Figure 2:**
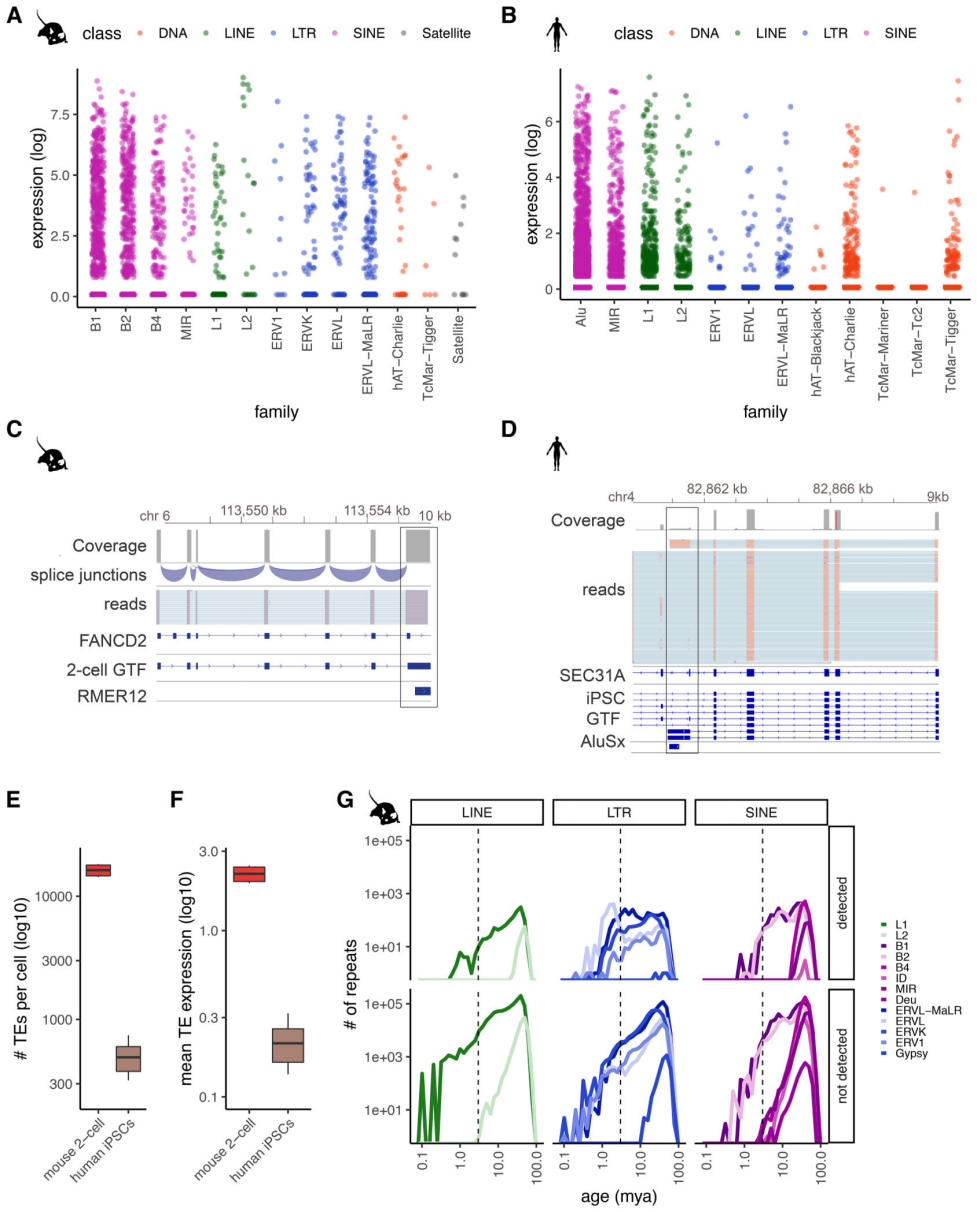
CELLO-seq enables TE-derived isoform and TE expression analysis in single cells at single loci. (A) Jitterplot of expression level of TE-derived isoforms in mouse blastomeres by repeat family and coloured by repeat class. B1 (n= 990), B2 (n=630), B4 (n=300), L1 (n=96), L2 (n=24), ERV1 (n=12), ERVK (n=90), ERVL (n=66), ERVL-MaLR (n=174), hAT-Charlie (n=36), TcMar-Tigger (n=6), Satellite (n=12). (B) Jitterplot of expression levelof TE-derived isoforms in hiPSCs by repeat family and coloured by repeat class. Alu (n=16643), MIR (n=4628), L1 (n=3649), L2 (n=1602), ERV1 (n=178), ERVL (n=445), ERVL-MaLR (n=534), hAT-Blackjack (n=89), hAT-Charlie (n=2225), TcMar-Mariner (n=89), TcMar-Tc2 (n=89), TcMar-Tigger (n=890). (C) IGV genome browser view of stranded reads overlapping a TE-derived isoform by RMER12 integration in an intron of FANCD2 in mouse blastomeres (n=6 cells), exonic reads = blue, introns = turquoise. (D) genome browser view of stranded reads overlapping a TE-derived isoform by AluSx integration into intron of SEC31A in hiPSCs (n=96 cells)(E) boxplot of number of autonomous TEs in each cell (y-axis) in mouse 2-cell blastomeres (red) or hiPSCs (brown). Mouse (n=6 cells), human (n=96 cells). (F) boxplot of mean expression of autonomous TEs in each cell (y-axis) in mouse 2-cell blastomeres (red) or hiPSCs (brown). Mouse (n=6 cells), human (n=96 cells). (G) frequency plot of number of repeats (y-axis) by age of TE (mya) (x-axis). TEs mapped to mouse blastomeres (upper panel) and all repeats from mouse UCSC repeatmasker annotation (lower panel) .TEs are grouped by TE class and coloured by TE family. The boxplots shown in E and F show the median, first and third quartiles as a box, and the whiskers indicate the most extreme data point within 1.5 lengths of the box.

TEs have long been known to give rise to alternative transcript start and end sites, resulting in the generation of isoforms when the TEs fall near the coding region of existing genes ^39^. Long-read sequencing protocols that enable capture of full-length transcripts, such as CELLO-seq, provide an ideal opportunity to probe their expression. Consistent with previous studies, we found expression of TE-derived isoforms, defined as transcripts of non-TE genes whose TSS or TES overlaps a TE, leading to an alternative end or start of the transcript isoform. Most TE-derived isoforms give rise to a TSS and, in both human and mouse, these are enriched for SINE elements (Extended Data Fig S2A-D). TE-derived isoforms are clearly visible from ERVL-MaLR elements - ORR1 and MT in mouse blastomeres, and Alu elements in hiPSCs ^4,6,40–42^ (Fig 2A-D, Extended Data Fig S2C,D). In the mouse 2-cell blastomeres, most TE-derived isoforms stem from SINE B1 and B2 elements (Fig 2A, Extended Data Fig S2D). DNA transposons also give rise to TE-derived isoforms in both datasets (Fig 2A,B, Extended Data Fig S2C,D).

We next wanted to study the expression of the autonomous TEs themselves. To start, following error correction, we mapped reads to unique TE locations in the genome that did not overlap any exons in the Gencode database (Methods). In total, we observed expression of ~10,000 and ~1,000 unique TE loci per cell in mouse blastomeres and hiPSCs, respectively (Fig 2E,F, Extended Data Fig S2E). We observed expression of individual TEs of different ages (Methods), including those belonging to young (defined as being < 2 mya) subfamilies of TEs, which was impossible using existing protocols (Extended Data Fig S1A,B). Nevertheless, we noted that very young TEs tended to be less frequently detected in our dataset (Fig 2G, Extended Data Fig S2E-G). Since such young TE families contain members with highly similar sequences, we hypothesized that this high sequence similarity was impeding our ability to uniquely map reads.

### Simulations show accurate expression analysis of young L1s

**Figure 3:**
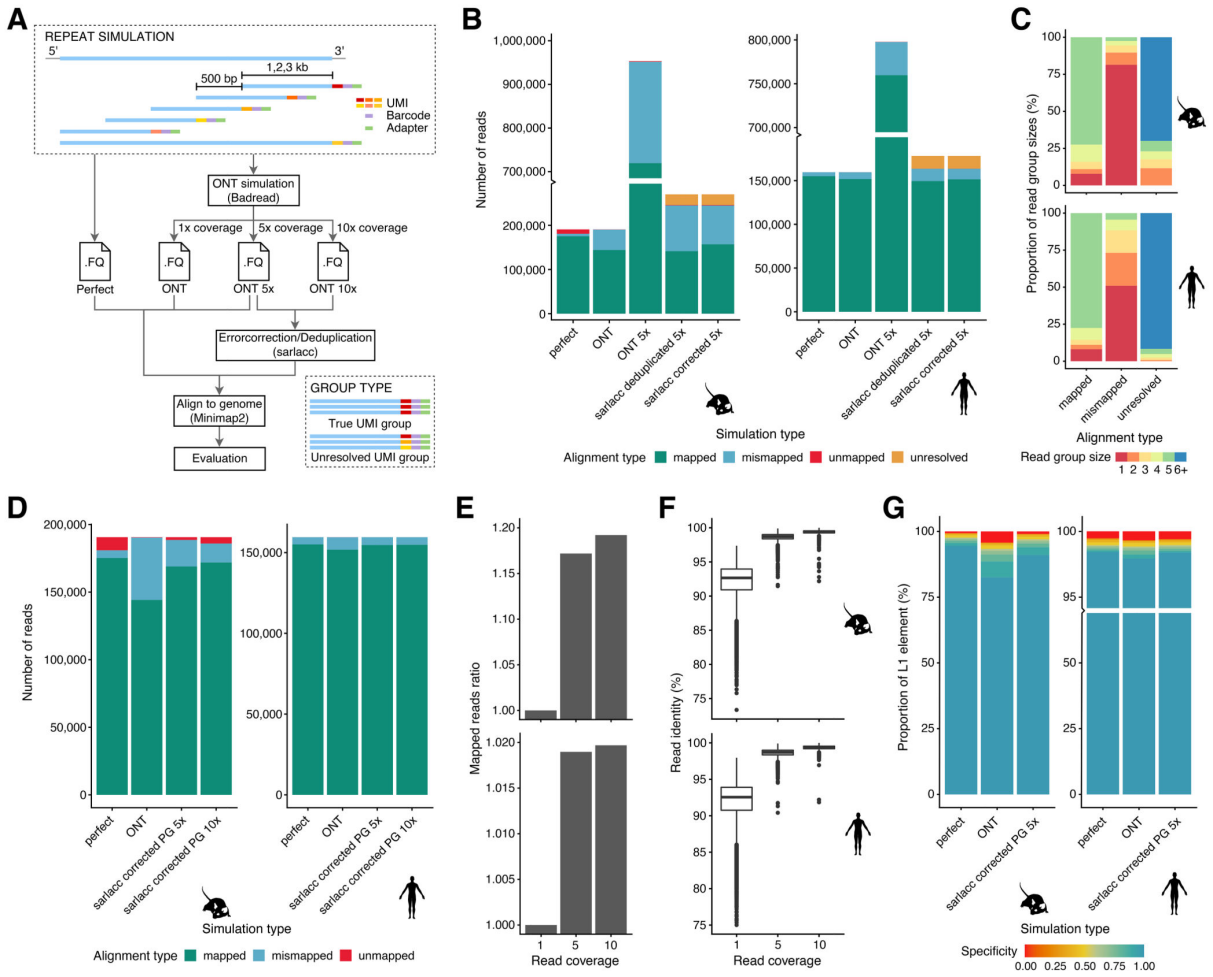
Simulations characterise the mapping of young L1 in mouse and human genome. (A) schematic of simulation of reads mapping to young L1s. We simulated reads in rolling windows from the 3’ end of young L1s for the human and mouse genome. We simulated 1-3 kb read length (data for 2kb reads shown) with 500bp gap between windows and 1x, 5x or 10x read coverage. We simulated reads with perfect or ONT read identity. Reads were directly mapped to the genome or processed using sarlacc to produce deduplicated and error corrected reads (Extended Data Fig S1D). (B) bargraph of number of reads (y-axis) by simulation type (x-axis), colour-coded by alignment type. Mapped = read at correct location after mapping to the genome, mismapped = reads mapped at wrong location, unmapped = read could not be mapped to the genome, unresolved = group with multiple molecules present in one group. mouse (left), human (right). (C) bargraph of proportion of read group sizes (y-axis) by alignment type (x-axis), colour-coded by group size; mouse (top), human (bottom). (D) stacked bargraph of number of reads (y-axis) by simulation type, coloured by alignment score. (E) bargraph shows ratio of mapped reads (y-axis) by read coverage (x-axis) for mouse (top) and human (bottom). (F) boxplot of read identity (y-axis) by read coverage (x-axis) for mouse (top) and human (bottom). Mouse read coverage: 1, 5, 10 (n=140147), human read coverage: 1, 5, 10 (n=149017). The boxplots show the median, first and third quartiles as a box, and the whiskers indicate the most extreme data point within 1.5 lengths of the box. (G) Stacked bargraph showing proportion of L1s(y-axis) by simulation type (x-axis), coloured by specificity score; mouse (left) and human (right). Simulation type: perfect = perfect read identity, ONT = ONT read identity, ONT 5x = ONT read identity with 5x coverage, sarlacc corrected 5x = ONT read identity score, 5x coverage with sarlacc error correction, sarlacc corrected 10x = ONT read identity score, 10x coverage with sarlacc error correction, sarlacc deduplicated 5x = ONT read identity score, 5x coverage with sarlacc deduplication by randomly choosing 1 read. PG = perfect grouping.

To investigate whether this lower detectability was driven by challenges in mapping arising from the high sequence similarity of such elements, we simulated reads in rolling windows from the 3’ end of all young L1PA and L1Md elements for the human and mouse genome, respectively. For each element, we simulated differing read length (1, 2 or 3kb reads - data for 1 and 3kb on github: https://github.com/MarioniLab/long_read_simulations) with either perfect or an ONT read identity error profile (Fig 3F, 1x coverage) ^43^, and considered data with 1x, 5x or 10x coverage of each read (Fig 3A).

To assess the ability to map reads to the correct locus, we considered three strategies – i) a naïve mapping of reads to the genome; ii) deduplication of reads with identical UMI sequence prior to mapping to the genome; and iii) error correction of reads with identical UMI sequence prior to mapping to the genome (Methods).

As expected, reads with an ONT read identity that were naïvely mapped to the genome were incorrectly mapped more often than perfect reads (Fig 3B, 1x and 10x coverage in Extended Data Fig S3A; perfect vs ONT). At 5x coverage, a naïve mapping of ONT reads led to a large (~5 times) increase in the number of mapped reads, artificially inflating the number of correctly and incorrectly mapped molecules. Our bespoke error correction or deduplication strategy substantially removed this artificial inflation of gene counts, but a substantial number of mismapped reads were still observed, especially for the mouse. Additionally, we noted that some grouped sets of reads were unresolved, being comprised of distinct molecules. Finally, we observed that error correction reduced the number of mismapped reads in comparison to deduplication (Fig 3B, Extended Data Fig S3A).

To understand what features might explain the set of reads that could not be properly mapped following error correction, we first compared the distribution of UMI counts in the mapped, mismapped and unresolved groups. For mapped reads, the UMI group sizes were generally equal to the read depth (5x), while mismapped and unresolved reads were grouped into smaller (<5x) and larger (>5x) groups, respectively (Fig 3C, 1x and 10x coverage in Extended Data Fig S3B). Since this suggested that imperfect grouping of UMI sequences might underpin the erroneously mapped reads, we next asked whether a perfect grouping would improve performance. Consistent with our hypothesis, perfect grouping would allow us to increase the proportion of correctly mapped reads with increased read identity score, especially for the mouse (Fig 3D-F). Finally, we investigated whether we could in principle optimise the grouping by generating reads with longer UMIs (Extended Data Fig S4).

To this end, we simulated differing UMI lengths (10-50) and differing read depth (5x and 10x) before assessing our ability to correctly group reads based on their UMIs using the Levenshtein Distance (S4A, Methods). When simulating data with an ONT error profile, we observed that 50nt UMIs were needed to correctly group the simulated data (Fig S4B-C). This contrasts with the relatively short (~10nt) UMI used for NGS and the 22nt UMI used in CELLO-Seq.

In sum, for the large majority of L1s in both human and mouse we determined that CELLO-seq reads could be correctly mapped, with the exception being very young mouse L1s due to difficulties in grouping reads (Extended Data Fig S3D). To avoid these loci confounding downstream analyses of our CELLO-seq data, for each L1 element we calculated a specificity score based on the number of mapped reads from the selected L1 element and the number of mismapped reads from other L1 element that aligned within the location of the selected L1 element (Table on GitHub: https://github.com/MarioniLab/long_read_simulations): 98% of the human L1s have a specificity score > 98% (with an increase to 98.5% for perfect sequence complementarity), while for mouse L1s, 80% of L1s have a specificity score > 80% with our current method (Fig 3G, 10x coverage Extended Data Fig S3C). Henceforth, we consider filtered datasets, only including L1s with a specificity score of 80% (Table S2).

### Unique TEs react differently to epigenetic reprogramming

**Figure 4:**
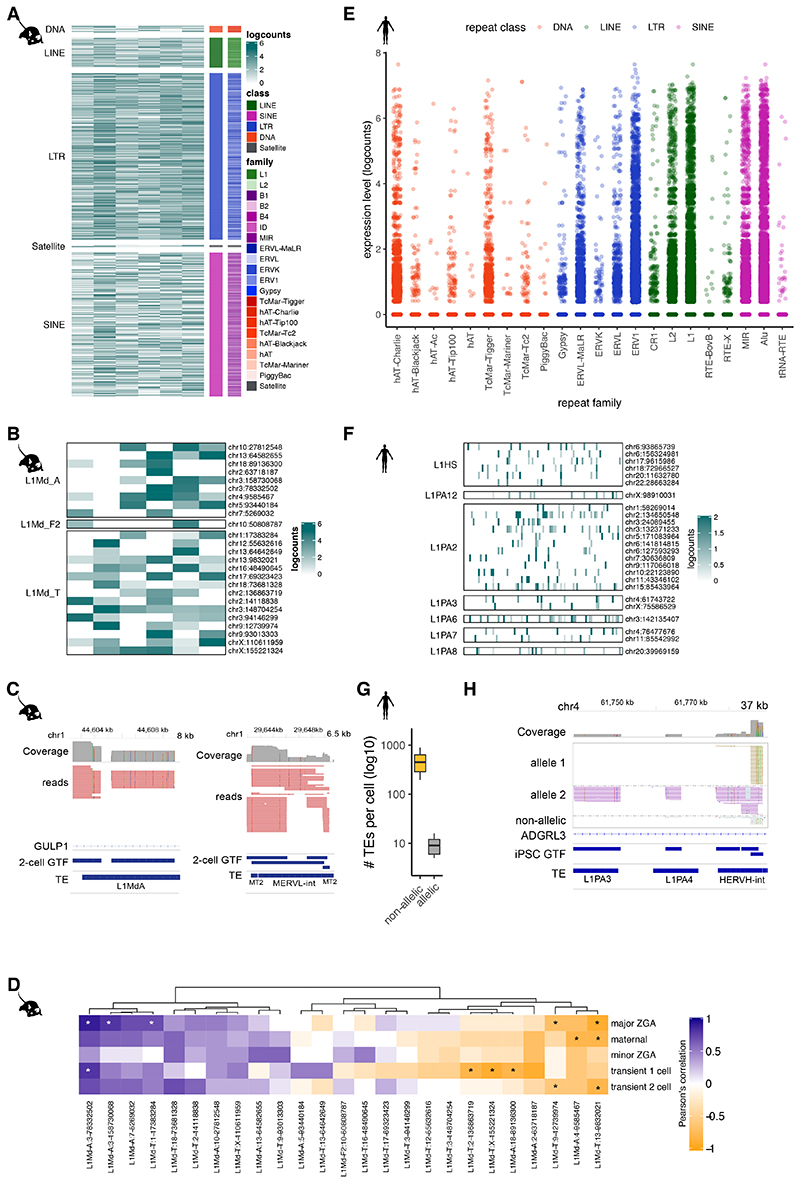
CELLO-seq enables study of young TEs at unique loci. (A) Expression of all highly expressed TEs (n=3618) (mean expression across all cells > 1 logcounts) in 2-cell mouse embryos. Clustering is performed by TE class. B1 (n=450), B2 (n=380), B4 (n=167), ERV1 (n=65), ERVK (n=282), ERVL (n=694), ERVL-MaLR (n=1159), hAT (n=1), hAT-Blackjack (n =2), hAT-Charlie (n=38), hAT-Tip100 (n=1), ID (n=2), L1 (n=285), L2 (n=19), MIR (n=35), PiggyBac (n=1), Satellite (n=20), TcMar-Mariner (n=1), TcMar-Tc2 (n=1), TcMar-Tigger (n=15). (B) Expression of all long (> 5 Kb) L1 elements that are highly expressed (mean >1 logcounts) in mouse 2-cell blastomeres clustered by their subfamilies. (C) genome browser view of stranded reads overlapping L1MdA (left) and MERVL-int (right) in mouse 2-cell embryos, sense reads = red. Shown are all reads from 6 2-cell blastomeres. 2-cell GTF has been created by FLAIR using CELLO-seq reads after error correction as input. (D) Pearson correlation of preimplantation networks with unique loci of young, long (>5.8 kb) L1 elements in mouse 2-cell blastomeres as a heatmap. (E) Expression of all TEs (n=414) in hiPSCs shown by family and coloured by TE class (sum of expression across all cells > 10 logcounts). hAT-Charlie (n=1335), TcMar-Tigger (n=534), TcMar-Tc2 (n=178), ERVL-MaLR (n=1157), ERVK (n=178), ERVL (n=534), ERV1 (n=3738), L2 (n=2937), L1 (n=9078), MIR (n=3827), Alu (n=12905). (F) Expression of all long (>5 Kb) TE elements that are highly expressed (mean >1 logcounts) in hiPSCs clustered by their subfamilies. (G) boxplot of mean number of TEs per cell (y-axis) which we can call allelically or non-allelically.The boxplots show the median, first and third quartiles as a box, and the whiskers indicate the most extreme data point within 1.5 lengths of the box. human (n=96 cells), mouse (n=6 cells). (H) genome browser view of stranded reads overlapping L1PA3, L1PA6 and HERVN-int in hiPSCs. Shown are all reads from 96 hiPSCs. iPSC GTF has been created by FLAIR using CELLO-seq reads after error correction as input. yellow = allele1 reads, purple = allele2 reads, grey = non-allelic reads. Significant associations (FDR < 0.1 = *).

During preimplantation development the whole genome is epigenetically reprogrammed, which in turn leads to substantial changes in the transcriptome. Consistent with previous studies, we observe a genome-wide TE expression in the sequenced mouse blastomeres at 2-cell stage embryos, with high expression of MERVL elements (Fig 4A, Extended Data Fig S5B,F,H)^4,40^. In contrast to SINE and MERVL elements, young full-length LINEs were consistently expressed from only a few loci (with only 0.3% of all genomic young L1s being expressed versus 5% of all MERVL being expressed) in all of the 2-cell blastomeres (Fig 4A-C, Extended Data Fig S5A,B,D,E Table S3). The young L1s had an intact open reading frames (ORFs) and low methylation level in preimplantation development (Extended Data Fig S6A,C, Table S4) ^44,45^.

Given the ability of CELLO-seq to detect locus specific TE expression, we had the opportunity to explore whether the expression of specific TEs is associated with the expression of different classes of protein coding genes and to infer putative regulatory mechanisms. Prolonged L1 expression is associated with the arrest of mouse preimplantation development ^26,27^. CELLO-seq allowed us to test the correlation between the expression of genes associated with different stages of preimplantation development and the expression of young L1s in mouse 2-cell blastomeres^46^. Consistent with previous work, the majority of L1 loci positively correlate with early development expression profiles. We also observed a subset of L1 loci with a statistically significant negative correlation, suggesting that these TEs might be relevant for the transition between 2-cell and gastrulation stages (Fig 4D). To test the biological relevance of this finding, a larger sample size and follow up experiments are required in order to investigate whether knockdown of specific L1s can lead to disruption of preimplantation development ^26,27^. These correlations show the relevance of studying TEs as unique insertions, as the genomic location in which the element is situated plays a role in the expression of the element.

CELLO-seq also enabled us to measure expression at unique young L1, Alu, and HERV-int elements in hiPSCs (Fig 4E, Extended Data Fig S5C). Coherent with previous findings in bulk and single cell NGS datasets ^6,41,42^ Alu elements contributed the highest number of expressed loci, while the most highly expressed TEs were HERV-int elements and their LTRs – LTR7 (Fig 4E,H, Extended Data Fig S5C,G,I). CELLO-seq permitted us to detect locus-specific expression of L1Hs and L1PA2s (Fig 4F,H, Extended Data Fig S6B,D, Table S3), some of which are known to be retrotransposition competent, have full-length ORFs and are demethylated in tumours and in blastocysts ^14,47–55^ (Extended Data Fig S6B,D, Table S4). Additionally, we were able to study allelic TE expression at single cell resolution, with a mean number of ten TEs showing allele-specific expression in each hiPSC (Fig 4G, H).

## Discussion

CELLO-seq is a tool for profiling the expression of full-length molecules, including transposable elements, in single cells. Unlike other approaches that have generated long transcript reads from single-cells, CELLO-Seq does not rely on library generation using the 10X Chromium, or other similar platforms, but rather generates libraries that can be immediately sequenced using ONT. As a result, in the development of CELLO-seq we have been able to optimise conditions for RT and PCR, and to link our oligonucleotide design to our bespoke analysis pipeline, all of which enable full-length molecules to be captured and quantified more reliably. A crucial feature of CELLO-seq is the ability to take advantage of PCR duplicates to error correct reads prior to mapping. Moving forward, we envisage that the efficiency of this error correction step could be improved by increasing the number of PCR duplicates for each read via target enrichment, or by performing emulsion PCR to inhibit the PCR jackpot effect, thereby flattening the distribution of PCR duplicates among UMIs (Extended Data Fig S1C).

Even with the innovations of CELLO-seq, challenges remain. For example, measuring expression at very young TEs is challenging due to their very high sequence similarity and for L1s most of the reads are concentrated on the 5’ end and do not cover the full-length transcripts. Using simulations, we demonstrated that while we could map L1s across the human genome with high specificity, mapping young mouse L1s to unique loci with high specificity is currently only possible for ~85% of loci. To study TEs with very high sequence identity, our simulations suggest that we will need to increase the UMI length in CELLO-seq to 50nt in order to enable accurate grouping of reads with ONT read identity error profile. Our simulations show that even 36bp UMIs as used in Karst et al., 2021^56^ might not be long enough to study young L1 elements, especially as the UMI is split across 18bp at 5’ and 3’ end of each molecule. We note that while a 50nt UMI is much longer than any used by existing single-cell technology, it would be feasible in CELLO-seq since we use a splint-ligation to add the UMI sequence. ONT sequencing of rolling circle amplification products has also been demonstrated as a means of delivering error-corrected transcript reads ^57,58^. However, the raw read throughput of these protocols is low compared to CELLO-seq. Advances in chemistry and basecallers provided by Oxford Nanopore Technologies will constantly improve the error rate, promising great promise for single cell long read technologies. In contrast, PacBio sequencing, which also exploits rolling-circle replication, can now deliver very high fidelity reads (~Q30) due to the many polymerase passes of each insert seen in recent chemistry upgrades. However, the unique read throughput remains constrained by the fixed number of zero mode waveguides manufactured on each flow cell, rendering this platform relatively expensive.

From a biological perspective, our data already demonstrate how CELLO-seq can be used to provide insight into the transcription and regulation of young TEs and opens the possibility to study polymorphic L1s. We have shown that expression of TEs is heterogeneous across members of a TE subfamily and, moreover, that members of the same subfamily are differentially correlated with the expression of putative regulatory elements. This presents the intriguing possibility that different classes of epigenetic regulators can impact the expression of unique TEs from the same family, potentially leading to stage-specific expression during development.

In conclusion, CELLO-seq enables isoform, allelic and TE expression at unique loci at single cell resolution. Our simulations show that we are able to map autonomous TEs with high specificity at most loci. The possibilities to study the role of transposable elements at unique loci – the same way we study protein coding genes - will provide insight into TE biology and the role of TEs in gene expression.

## Methods

### Cell culture

#### Human-induced pluripotent stem cells

Human-induced pluripotent stem cells (hiPSCs) (https://www.hipsci.org/lines/#/lines/HPSI0714i-nufh_3, https://www.hipsci.org/lines/#/lines/HPSI0914i-euts_1)^59^ were thawed and cultured under feeder-free conditions on Vitronectin XF™-coated (Stem Cell, #07180) 10 cm tissue culture treated plates (Corning) in complete Essential 8 medium (Life Technologies #A1517001) supplemented with 1% Penicillin/Streptomycin (Invitrogen, #15140122). Cells were incubated at 37°C, 5% CO2 and media changed every day except for the day of passaging. Cells were routinely passaged using 0.5 mM EDTA (Life Technologies #AM9260G) at least 3 times post-thawing before collection. HiPSCs were harvested using Accutase (Millipore, #SCR005) to generate a single cell suspension. Single cells were resuspended in 1X PBS (Thermo Fisher Scientific, #10010023) and passed through a 40uM filter (Corning #CLS431750-50EA) before FAC sorting (BD Influx™ Cell Sorter) into 96-well plates (Framestar, #4TI-0960) containing 2 μl lysis buffer with 1 U/μl Rnase Inhibitor (RRI) and ERCC spike in mix at a 1:20 dilution (Ambion Cat #4456740). Plates were promptly sealed (Thermo Fisher Scientific #AB0626), spun down and frozen at -80 °C until further processing.

#### Mouse embryonic stem cells in 2i medium

Male E14 mouse embryonic stem cells (mESCs) were maintained in culture with Dulbecco’s Modified Eagle Medium (ThermoFisherScientific, #41965062) supplemented with 10 % fetal calf serum (ThermoFisherScientific), 2 mM L-glutamine (ThermoFisherScientific, #25030081), 0.1 mM non-essential amino acids (FisherScientific, #11140035), 50 μM β-mercaptoethanol (ThermoFisherScientific, #31350010), 100 U/ml penicillin/ 100 μg/ml streptomycin (ThermoFisherScientific, #15140122) and 1000 U/ml LIF (made in-house). Cells were grown without feeder cells on gelatin-coated plates under standard culture conditions (37 C, 5% CO2, humid) and passaged upon ~80 % confluency every 2-3 days using TrypLE Express (ThermoFisherScientific, # 12604013).

Conversion to ground state pluripotency was performed by switching to 2i serum-free growth media comprised of N2B27 media (50:50 DMEM/F-12 (ThermoFisherScientific #11320033):Neurobasal (ThermoFisherScientific, #21103049) supplemented with 1 x N2 (ThermoFisherScientific, #17502048), 1 x B27 (ThermoFisherScientific, #17504044), 2 mM L-glutamine, 50 μM β-mercaptoethanol, 100 U/ml penicillin/100 μg/ml streptomycin (all from ThermoFisherScientific) and 1000 U/ml LIF (made in-house)) with addition of 3 μM Gsk3 inhibitor (CHIR99021, Sigma Aldrich, # SML1046-5MG), 1 μM MEK inhibitor (P00325901, Sigma Aldrich, #PZ0162-5MG), and 0.1 mg/ml Vitamin C (Merck, #V-047-1ML). In 2i conditions, extra care was taken to minimise loss of cells during passaging, which was performed every 3 days by Accutase (Sigma Aldrich, #A6964-100ML) treatment followed by centrifugation (437 x g, 5 minutes) in PBS (ThermoFisherScientific, #20012068) and re-plating, at 1/2-1/4 dilution in 2i serum-free growth media, into plates that had been pre-gelatinised for 30 minutes at 37 C.

On the day of cell sorting, cells were harvested with Accutase treatment followed by two PBS washes of the cell pellet, then passed through a 40 μm strainer (Falcon, #352340) and left on ice as 500 μl of single cells in suspension in PBS. 1 μg/μl DAPI (ThermoFisherScientific, #D1306) was added prior to sorting of live cells into single wells of 96-well plates (Framestar, #4TI-0960) filled with 2 μl CELLO-seq lysis buffer using a BD Aria III machine (Becton Dickinson) at the WIMM Flow Cytometry Facility.

### Collection of mouse preimplantation embryos

#### Ethical considerations

All experimental procedures were in accordance with UK Home Office regulations and the Animals (Scientific Procedures) Act 1986 (PPL No: P418B15F6). All experimental protocols were approved by the Animal Welfare and Ethical Review Body (AWERB) of the University of Cambridge CRUK Cambridge Institute. At the end of the study, mice were euthanized by cervical dislocation, in accordance with the above stated UK Home Office regulations.

#### Reagents

Human chorionic gonadotropin (Chorulon; National Veterinary Services, #804745), Pregnant mare serum gonadotropin (PMSG; National Veterinary Services, #859448), EmbryoMax KSOM medium with 1/2 amino acids without BSA (Reagent setup; Merck Millipore, cat. no. MR- 107-D), BSA powder (Sigma-Aldrich, cat. no. A3311), M2 medium with HEPES (Sigma-Aldrich, cat. no. M7167), Cytochalasin B (Reagent setup; Sigma-Aldrich, #C6762), Tyrode’s solution, acidic (Sigma-Aldrich, #T1788), Dimethyl sulfoxide (DMSO; Sigma-Aldrich, #D2438).

Supplemented EmbryoMax KSOM medium: Before separation of blastomeres from embryos, further supplement 50.0 mL of EmbryoMax KSOM medium with 20.0 μL of phenol red solution (0.5% (wt/vol) in H2O) and 3.0 mg/mL BSA. The medium should be prepared in a sterile tissue culture hood and can be stored at 4 °C for 2 weeks. Warm the medium in a 37 °C, 5% CO2 incubator for at least 30 min before use.

Cytochalasin B stock solution: Prepare a stock solution of 1.0 mg/mL cytochalasin B by resuspending 1.0 mg of cytochalasin B in 1.0 mL of DMSO. Divide the solution into single-use aliquots, 7.5 μL each, and store at –20 °C for up to 6 months.

#### Collection of blastomeres from embryos

To collect individual blastomeres from two-cell stage mouse embryos, female C57BL/6J mice older than 8 weeks of age were super-ovulated by intraperitoneal injection of pregnant mare serum gonadotropin (7.5 IU per mouse) at 4PM. 46–48 h later (i.e., 2–4 pm), each mouse was injected with 7.5 IU of human chorionic gonadotropin and left to mate with proven studs (C57BL/6J). Vaginal plugs were checked the following morning (0.5 d.p.c.) and mice that have successfully mated were used for dissection.

For late 2-cell stage embryos collection, the plugged mouse was culled at 4:30 pm, and the uterine horns and oviducts of the donor mouse were dissected under a dissection microscope and placed on the lid of a 100-mm tissue culture dish in M2 medium. The oviduct with part of the uterine horn was cut out of the uterus and place it into M2 medium. Under a dissection microscope, a needle was inserted infundibulum and the oviduct was flushed with M2 medium, with late 2-cell stage embryos being flushed out into a dish. The embryos were cultured in 1.0 mL of supplemented EmbryoMax KSOM medium with phenol red (containing 3.0 mg/mL BSA) in a 5% CO2 humidified incubator at 37 °C. Just prior to separating the blastomeres, embryos were cultured in 1.0 ml of supplemented EmbryoMax KSOM medium containing 3.0 mg/mL BSA and 7.5 μg/mL cytochalasin B for at least 30 min. A dish with four drops of M2 medium with 7.5 μg/mL cytochalasin B and two drops of Tyrode’s solution was prepared. Under a dissection microscope, the embryos were transferred to M2 medium with 7.5μg/mL cytochalasin B. All embryos were transferred into the first drop of Tyrode’s solution (washing step) and then into the second drop of Tyrode’s solution. We waited until the zona pellucida of the embryos had dissolved, and quickly transferred the embryos into the first M2 drop, and washed the embryos through the second and third M2 drops. The blastomeres were transferred to an M2 drop immediately to minimize the damage of the acidic Tyrode’s solution to blastomeres. A thin capillary pipette with a blunt end was used to aspirate and blow out the blastomeres a few times to dissociate them. The individual blastomeres were then transferred into the fourth M2 drop, where they remained (for up to 30 min) prior until further processing.

Single cells were subsequently mouth pipetted into 96-well plates (Framestars #4TI-0960) containing 2 μL lysis buffer with 1 U/μL Superase In (Thermo Fisher Scientific, #AM2694), ERCC spike in mix at 1 in 20Mio dilution (Ambion #4456740). Plates were sealed (Thermo Fisher Scientific #AB0626) immediately spun down and frozen at –80 °C until further processing.

### CELLO-seq

Before starting with reverse transcription, we first eliminated any secondary structure in the RNA. We added a 2.2 μl mastermix of 1 μl (1mM) dT-oligo (IDT), 1 μl (1μM) dNTPs (25 mM each, Thermo Fisher Scientific #R0182) as well as 0.2 μl (20U/μL) of RNAse inhibitor (Superase In, Thermo Fisher Scientific, #AM2694) and incubated the mix at 72°C for 3min before returning to ice. The reverse transcription was performed in 10 μl preparing a 5.8 μl mastermix with 1 μl of 200U/μl Superscript IV Reverse Transcriptase (Invitrogen #18090050), 2 μl of 5x Superscript IV RT buffer, 1 μl of 5M betaine, 0.1 μl of (100μM) template switch oligo (IDT), 0.5 μl of 100mM DTT, 0.1 μl ET SSB (NEB #M2401S) and 1.6 μl nuclease free H2O (DNase/RNase-Free Distilled H2O, Thermo Fisher Scientific #10977-049). cDNA synthesis and template switching were performed for 10 min at 57 °C and 120min at 42 °C. After RT we digested all residual oligos using adding 5 μl mastermix of 4 μl nuclease free H2O (DNase/RNase-Free Distilled H2O, Thermo Fisher Scientific #10977-049), 0.5 μl of Exonuclease I (NEB #M0568) in 0.5 μl of 10x Cutsmart buffer (NEB # B7204S). We incubated the reaction at 37 °C for 1h, followed by 95 °C for 3 min to fully denature both the exonuclease and cDNAs. Before performing the splint ligation, we annealed the splint oligos in 1 μl of 10x Oligo hybridisation buffer (500 mM NaCl, 10 mM Tris-Cl, pH 8.0, 1 mM EDTA, pH 8.0), 1 μl of 100 μM top splint (IDT) and 1 μl of 100 μM bottom splint (IDT) diluted in 7 μl nuclease free H2O (DNase/RNase-Free Distilled H2O, Thermo Fisher Scientific #10977-049). The reaction was annealed at 95 °C for 1m following 20 s at 95 °C and decreasing temperature by 1 °C every cycle, this step was repeated 80 times and subsequently kept at 4 °C until further processing.

The splint ligation reaction was performed by preparing a 5 μl mastermix that consisted of 0.5 μL Hifi Taq Ligase (NEB #M0647S), 0.5 μl 10x Taq ligase buffer, 1μl of 10 μM annealed splint oligo, 0.25 μL NAD+ (NEB #B9007S) and 2.75 μl nuclease free H2O (DNase/RNase-Free Distilled H2O, Thermo Fisher Scientific #10977-049). The ligation was performed at 55 °C for 1h. Before clean-up we performed a Proteinase K digestion, as the beads were sticky with leftover protein. We digested the proteins with Proteinase K (NEB #P8107S) for 5 min at 50 °C. Barcoded cDNA was then pooled in 2 mL DNA LoBind tubes (Eppendorf #0030108051) and cleaned up using AMPure XP beads at a ratio of 0.5X volume. Purified cDNA was eluted in 12.75 μl H2O. 0.75 μl of the purified DNA was used to perform a proxy qPCR to avoid over-amplification. For the qPCR we used a 25 μL reaction with 12.5μl of 2x KAPA HiFi Uracil+ hot start polymerase mix (Roche # KK2800), 1.25μl of 10 μM forward and reverse PCR primers (IDT), 5μl of 5M betaine, 0.5 μL ET SSB, and 1.25μl of 2x EvaGreen (Biotium #31000-T). The qPCR was performed for 3 min at 90 °C for initial denaturation followed by 29 cycles of 20 s at 90 °C, 30 s at 65 °C, 10 min at 72 °C for 40 cycles. We then used four times fewer cycles than indicated by the qPCR to lead to logarithmic phase of amplification and prepared a PCR reaction of 50 μl PCR master mix consisting of 25μl of 2x KAPA HiFi Uracil+ hot start polymerase (Roche # KK2800) mix, 2.5μl of 10 μM forward and reverse PCR primer, 10μl of 5M betaine, 0.5 μL ET SSB. The PCR was cycled as given: 3 min at 90 °C for initial denaturation followed by 29 cycles of 30 s at 90 °C, 15 s at 65 °C, 10 min at 72 °C, for 25-29 cycles, depending on the qPCR results. These high cycle counts are required to yield the high cDNA inputs (>500 ng) required for optimal ONT sequencing. Final elongation was performed for 10 min at 72 °C. Following amplification, all samples were purified using AmpureXP beads at a volumetric ratio of 0.5X with a final elution in 13 μl of H2O (DNase/RNase-Free Distilled H2O, Thermo Fisher Scientific #10977-049). The cDNA was then quantified using the Qubit High Sensitivity dsDNA Assay Kit (Thermo Fisher Scientific, #Q32851). Size distributions were checked on High-Sensitivity DNA chips (Agilent 2100 Bioanalyzer, #5067-4626). All oligo sequences can be found in Table S5.

### Smart-seq2

We prepared cDNA of human iPSCs following the Smart-seq2 protocol ^60^.

### ScNaUMI-seq

We prepared cDNA of mESCs following the ScNaUMI-seq protocol, loading 800 cells on one 10x flow cell ^28^.

### Library preparation and sequencing

#### Short read library prep from CELLO-seq derived cDNAs

For tagmentation we used custom made Tn5 provided by the Protein Expression and Purification Core Facility of the European Molecular Biology Laboratory (EMBL). We subsequently largely followed the previously published protocol with small adjustments^61^. We first annealed the oligos in Oligo hybridisation buffer (500 mM NaCl, 10 mM Tris-Cl, pH 8.0, 1 mM EDTA, pH 8.0), and we used Tn5MErev ^62^ and Nextera R1 oligo (IDT, Table S5) at a final concentration of 350 μM in total 5.15μl H_2_O. The reaction was annealed at 95 °C for 1 min following 20 s at 95 °C and decreasing temperature by 1 °C every cycle; this step was repeated 80 times and the resulting material kept at 4 °C until further processing. We then loaded the annealed oligo onto the Tn5 using 35 μM annealed oligo in 10μl Tn5 with 0.5μl Oligo Hybridisation buffer (500 mM NaCl, 10 mM Tris-Cl, pH 8.0, 1 mM EDTA, pH 8.0). We incubated for 30min at 23 °C. The tagmentation was performed in 10 mM Tris-HCl pH 7.5 (Merck, #93363), 10 mM MgCl_2_ (Merck, #M1028), and 25% dimethylformamide (DMF) (Merck, # 227056) using 200 pg target DNA and incubated at 55 °C for 3 min in a preheated thermocycler. To unbind the Tn5 we performed a proteinase K digestion using 0.5 μl proteinase K in 1μl yeast tRNA in 10 mM Tris-HCl pH 7.5 and incubated for 5min at 55 °C. The product was purified using SPRI beads at a ratio of 1:1. We eluted in 12.75μl H2O and used 0.75μl to perform a proxy qPCR in order to not overcycle. The qPCR was performed as above using KAPA HiFi hot start polymerase and Illumina i5 and i7 primers (Table S5). We included a 3 min 72 °C step before starting the initial denaturation, annealed at 67 °C and performed the elongation for 30 s instead of 10 min. We then used 6 times less cycles than the qPCR indicated to perform the PCR as stated above using KAPA HiFi hot start polymerase and Illumina i5 and i7 primers. We included a 3 min 72 °C step before starting the initial denaturation, annealed at 67 °C, performed the elongation for 30 s instead of 10 min and performed 11 PCR cycles. The product was bead purified at 0.6X. The libraries were checked on an Agilent 2100 Bioanalyzer and quantified using Qubit as well as a KAPA library quantification kit, as per manufacturer’s recommendations. Libraries were paired-end sequenced on an Illumina HiSeq 2500 instrument.

#### Short reads for Smart-seq2

Amplified cDNA produced by Smart-seq2 was, in lieu of the Nextera XT tagmentation recommended in the protocol as written, fragmented and processed into an Illumina sequencing library using the NEB Ultra II FS kit (NEB cat. no. E7805S). Libraries were paired-end sequenced on an Illumina HiSeq 4000 instrument.

#### Short reads for ScNaUMI-seq

Half of the pre-amplified cDNA produced by ScNaUMI-seq was processed into an Illumina sequencing library using the NEB Ultra II FS kit (NEB cat. no. E7805S). Libraries were paired-end sequenced on a NovaSeq 4000 instrument. The other half was further amplified according to the ScNaUMI-seq protocol, ONT sequencing libraries were prepared using the Ligation Sequencing Kit (ONT SQK-LSK110) and sequenced using three MinION R9.4.1 flow cells.

#### Long reads

200 fmol of CELLO-seq libraries were prepared for ONT sequencing using the Ligation Sequencing Kit (ONT SQK-LSK109) and sequenced using one MinION R9.4.1 flow cell for human iPSCs and mouse 2cell blastomeres. 200 fmol of CELLO-seq libraries were prepared for ONT sequencing using the Ligation Sequencing Kit (ONT SQK-LSK110) and sequenced using three MinION R9.4.1 flow cell for mESCs.

### Primary data processing

#### Short reads

The human iPSCs Smart-seq2 fastq data were processed with FilterByTile from the BBMap (38.76) ^63^ package, Trim Galore (0.6.5) ^64^ and GATK (4.1.4.0) ^65^ for filtering of low quality reads, adapter removal and deduplication, while the mouse 2-cell blastomeres fastq data were processed using UMItools^66^ (1.0.0) for demultiplexing and deduplication. STAR (2.7.3a) was then used for alignment to generate expression profiles for barcoded UMI data ^66,67^. For human iPSCs, we mapped to the human reference genome (gencode, GRCh38_p13) while mouse cells were mapped to the mouse genome (gencode, GRCm38_p6) concatenated with the ERCC reference.

We used featureCounts (2.0.0) for generating genic count data from the reads, with gencode GRCh38_p13 for human and GRCm38_p6 for mouse as annotation ^68^. After initial data processing, we filtered cells by spike-in normalisation, total library counts and mitochondrial RNA contamination following the Orchestrating Single-Cell Analysis with Bioconductor pipeline ^69,70^.

#### Long reads

After MinION sequencing, we basecalled the raw ONT reads using the high-accuracy model implemented in Guppy (3.5.2) and then preprocessed the reads using the sarlacc pipeline (Extended Data Fig S1H) ^71^. We first checked for internal adapters, which could arise from blunt ligation during ONT library preparation, and used Porechop (0.2.4) ^72^ to split the reads if more than 20% of reads had internal adapters. Otherwise, we filtered out reads with internal adapters present. We then performed adapter quality control, where we aligned the 250 bp sequence on either end of the read against dT-oligo and TSO sequences to identify the adapters at either end of the full-length cDNA, followed by filtering for reads with both adapters present^73^. We demultiplexed the samples based on the sample barcodes by grouping barcodes that have a Levenshtein distance below the grouping threshold. To reduce the search space for the UMI grouping, we performed pre-grouping by mapping the reads to the relevant transcriptome (defined above), ERCC spike-ins as well as unique genome repeat locations of mouse and human UCSC RepeatMasker (3.0) ^74,75^ track with minimap2 (2.17-r954-dirty) ^73^. We grouped the reads by their UMI sequence within each pregroup and either error corrected the reads by taking the consensus sequence from multiple sequence alignment of up to 50 reads in the UMI group, or by picking a random read from the UMI group in deduplication mode. For this study we used error corrected reads and aligned them back to the genome with minimap2.

#### Comparison of ScNaUMI-seq and CELLO-seq

The mESC ScNaUMIseq Illumina library were processed using the 10x Genomics Cell Ranger platform (5.0.1). Reads were aligned to the mouse reference genome (ENSEMBL, GRCh38 release 98) and quantified using either gencode mouse GRCm38_p6 annotation or an intergenic transposable element annotation, containing only transposable elements from the mouse UCSC repeat masker annotation that do not overlap with any element from gencode mouse GRCm38_p6 annotation.

Droplets containing captured cells were then called using the emptyDrops function from the DropletUtils^76^ (3.13) R package, using a UMI threshold of 5000 and an FDR of 0.001 on the genic count data. However, the number of droplets identified were higher than the number of cells loaded due to the very low input cell number. We therefore selected the valid cell containing droplets from the emptyDrops called droplets based on the total UMI content to match the number of cells loaded on the 10x flow cell. We processed the count data to filter for young L1 elements by filtering for L1 elements >5.8kb length and plotted the 50 highest expressed L1 elements in 2i grown mESCs.

#### Expression and epigenetic modification of MERVL and HERVL-int in mouse and human bulk data

We used Trim Galore to filter low quality reads and trim adapters, and STAR to uniquely map short read data of published RNAseq datasets for mouse 2-cell stage embryos and human iPSCs to the mouse genome (gencode, GRCm38_p6) and human reference genome (gencode, GRCh38_p13), respectively. Reads were quantified with featureCounts using mouse or human intergenic transposable element annotation, containing only transposable elements from the UCSC repeat masker annotation that do not overlap with any element from gencode annotation GRCm38_p6 or GRCh38_p13 for mouse or human, respectively. For mouse 2cell data, we defined high confidence MERVL elements by filtering for MERVL > 5kb length with more than 1 read in either dataset ^44,77,78^. We used bedtools (2.29.2) ^79^ intersect to overlay these expressed elements with uniquely mapped peak data of histone enrichment (H3K4me3, H3K27me3, H3K9me3), ATACseq peaks or DNA methylation level ^44,77,78,80,81^. For human iPSCs we defined high confidence HERVH-int elements in previously published RNAseq data by filtering for elements >3kb length and with at least 10 reads overlapping HERVH-int in NUFH3 RNAseq data^59,82,83^. We used bedtools intersect to overlay these high confidence HERVH-int elements with uniquely mapped peak data of histone enrichment (H3K4me3, H3K27me3), ATACseq peaks or DNA methylation level in previously published datasets ^47,83,84^.

#### Mobility of L1s

We used UCSC genome browser to obtain the DNA sequence of expressed L1 elements in human and mouse genome defined by CELLO-seq (Fig 4B,F). We then used ORFfinder (0.4.3)^85^ to check for full-length ORFs in these L1s. For human, we additionally searched for reference L1s that have previously found to be mobile (Table S4)^14,49–54^. In order to study whether the young L1s which are found to be transcribed by CELLO- seq are becoming demethylated at any stage during development or in tumour samples, we used bed intersect to overlay the previously published DNA methylation calls of mouse preimplantation development , human tumour samples, human iPSCs or preimplantation development with the expressed L1s in 2cell or human iPSCs.

#### Allelic gene expression analysis

We lifted over the phased vcf files from the Hipsci consortium using liftOver from UCSC. We then aligned the corrected reads against the genome using minimap2 and ran whatshap (0.18) to phase the reads^86^. The phased bam files were used in the isoform analysis.

#### Isoform expression analysis

To acquire splice-junction corrected isoform data, we corrected the reads using FLAIR (Full-Length Alternative Isoform analysis of RNA, 1.4.0) ^38^. The reads were first mapped to the transcriptome with repeat sequences and ERCCs added using flair-align. We then run flair-correct to perform splice-junction correction by using short read data for the human and mouse genome. During flair-collapse, the reads are grouped by their isoform and are corrected within each isoform separately. Afterwards, we used flair-quantify with salmon to generate isoform count data for each cell . We defined known isoforms as those with known ensembl transcript ID. Any isoforms lacking a known transcript ID were further subdivided into those overlapping repeats, termed TE-derived isoforms, or those that did not overlap a RepeatMasker entry, termed novel isoforms. To define TE-derived isoforms, we filtered the flair derived isoform GTF file using bedtools by considering only those isoforms whose transcription start site or transcription end site fell within a repetitive element, defined using the RepeatMasker annotation and the nested repeat file downloaded from UCSC.

#### Transposable element expression analysis

For autonomous transposable element analysis, we downloaded RepeatMasker and nested repeat annotation from UCSC in GTF format. For the final flair alignment, we used the transcriptome and repeat GTF to quantify TE-derived isoforms as well autonomous TEs.

#### Read normalisation

We used the isoform count data from flair-quantify and then filtered cells by spike-in normalisation, total library counts and mitochondrial RNA contamination following the Orchestrating Single-Cell Analysis with bioconductor (3.10) pipeline in R (3.6.3).

#### Transposable element age analysis

The age of transposable elements was extracted by converting the milliDivergence of each transposable element from the RepeatMasker annotation into million years of age using the Jukes-Cantor model.

#### CELLO-seq reads in representative genomic loci

We selected representative regions in the genome to show reads overlapping TE-derived isoforms, allelic TE expression, locus-specific TE expression as well as reads mapping to L1 loci using the IGV genome browser (2.7.2) ^88^.

### Repeat simulation

For repeat simulation, we obtained repeat sequences of all young L1PA and L1Md elements with length >= 2kb from the UCSC repeatmasker track. We then generated simulated reads with Python (3) using a rolling window of size 1, 2 and 3 kb, and gap size of 500 bp starting from the 3’ end of the L1 elements to better simulate the type of reads captured by CELLO-seq. For perfect simulation, we considered Illumina reads as our perfect baseline and therefore assigned Illumina high quality score (Q40) for each of the simulated reads, while for ONT simulation, we used a modified version of Badread to simulate ONT read identity and read coverage of 1x, 5x or 10x for each of the simulated reads. The identity parameter used for Badread simulation is 92,96,2.5 – corresponding to a mean read identity of 92%, maximum read identity of 96% and standard deviation of 2.5% – based on the read identity distribution of our ONT reads. We also used a custom error model and qscore model for Badread simulation, which are both trained from our ONT data. To allow for deduplication and error correction of the ONT simulated reads, we included the 3’ adapter used by CELLO-seq containing a shared cell barcode and unique simulated 22 bp RYN UMI for each of the simulated reads.

To assess the ability to map the simulated reads to their valid loci, we first processed the ONT simulated reads with 5x and 10x coverage using the sarlacc pipeline in order to produce both deduplicated and error corrected reads. We modified the sarlacc pipeline for the repeat simulation by aligning the simulated reads to the repeat sequences of the young L1PA and L1Md elements in the alignment stage. We then aligned the perfect simulated, ONT simulated and sarlacc processed reads against the reference genome using minimap2 and evaluated the location of the aligned reads against the true location of the simulated reads, which are stored in the simulated reads’ name.

We classified the reads into three categories based on the alignment type - mapped, for reads whose alignment location overlaps the true location by at least 1 bp; mismapped, for reads whose alignment location does not overlap the true location; and unmapped, for reads which did not get aligned. We also added an additional category for sarlacc processed reads - unresolved - for read groups formed by the sarlacc pipeline that are composed of multiple UMIs. We then summarise the information from each read at the L1 element level to calculate the fraction of reads that are mapped, mismapped and unmapped. We also calculated the specificity score for each L1 element based on the ratio of mapped reads from the selected L1 element and the number of mismapped reads from other L1 element that aligned wrongly within the location of the selected L1 element.

For read identity calculation, we ran minimap2 with -c flag in order to generate a CIGAR string for each alignment. The read identity for each read is then calculated based on the number of bases from the read sequence that matches the reference sequence and the number of bases from the read sequence that either does not match (mismatch) or is missing from the reference sequence (insertion/deletion).

We have published the pipeline used for repeat simulation, alongside the result of the young L1PA and L1Md element simulation for 1 and 3 kb reads, in GitHub (https://github.com/MarioniLab/long_read_simulations) to allow for simulation evaluation for other repeat types.

### UMI simulation

For UMI simulation, we generated 10,000 UMI-only reads of length 10, 20, 30, 40 and 50 bp with RYN or NNN pattern with Python (3). We then performed perfect simulation of the UMI reads with perfect read identity score at a read coverage of 1x, 5x and 10x, as well as ONT simulation using Badread to generate UMI reads with ONT read identity and read coverage of 1x, 5x and 10x, as described previously (data for 1x and 10x read coverage on Github, https://github.com/MarioniLab/long_read_simulations). Levenshtein distances were calculated across all true UMI pairs of perfect simulated UMI with 1x read coverage using the expectedDist function of the sarlacc R package. Group size and group purity evaluation were performed on the UMI groups produced by the umiGroups function of the sarlacc R package with the Levenshtein distance threshold for grouping set to 2, 6, 10 and 14. For no pregrouping evaluation, no pre-grouping of UMI were provided to the umiGroups function, while in pregrouping evaluation, the UMIs were first pre-assigned into groups of 100 unique UMI based on the true UMI sequences and this pre-grouping information was provided to the umiGroups function.

### Reporting Summary

Further information on research design is available in the Nature Research Reporting Summary linked to this article

### Code availability

For data analysis the code is available in the following GitHub repositories: https://github.com/MarioniLab/CELLOseq, https://github.com/MarioniLab/sarlacc, https://github.com/MarioniLab/long_read_simulations.

## Extended Data

**Extended Data Fig. 1:**
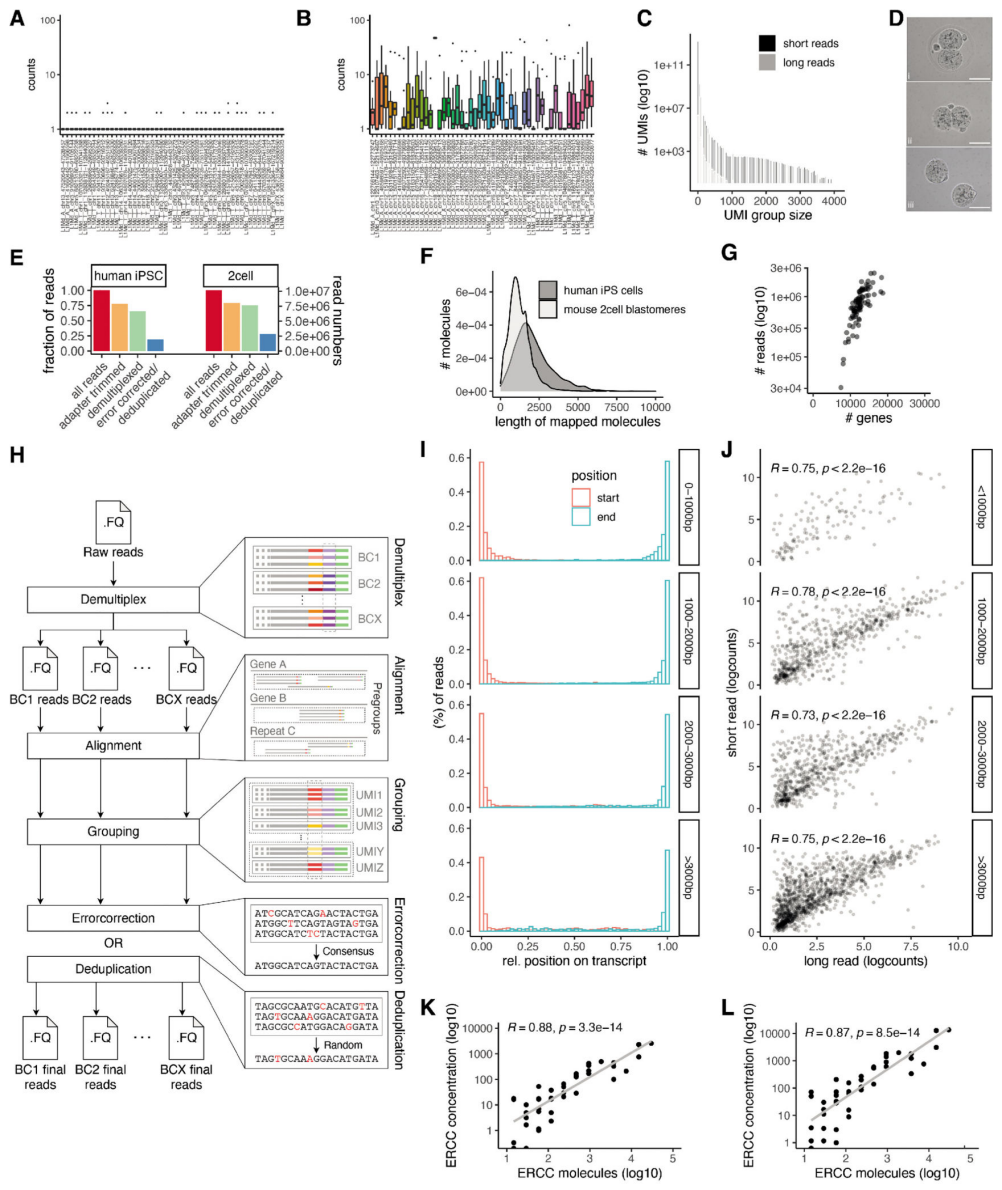
CELLO-seq library properties. Boxplots of counts across the 50 highest expressed young L1 unique loci in mouse ES cells cultured in 2i medium measured by (A) ScNaUmi-seq or (B) CELLO-seq. (C) histogram of number of UMIs (y-axis) by UMI group size (x-axis) for 2-cell blastomeres CELLO-seq data sequenced on a MinION flow cell or Illumina platform. (D) Light field microscopy image of two-cell embryo blastomere isolation. Two-cell embryo (i) with zona pellucida; (ii) without zona pellucida; (iii) as single blastomeres. This experiment was repeated more than 20 times. Scale bar 25μm. (E) bargraph of read numbers for mouse 2-cell embryo dataset (right) and human iPSCs (left). (F) density plot of number of molecules (y-axis) by length of mapped molecules (x-axis) for hiPSCs and mouse blastomeres. (G) scatter plot of number of reads (y-axis) versus number of genes in hiPSCs from Smart-seq2 libraries sequenced by Illumina. (H) schematic of sarlacc workflow. We demultiplexed samples by grouping barcodes with a Levenshtein distance below the grouping threshold. WE performed pre-grouping by mapping the reads to the relevant transcriptome. We grouped the reads by UMI sequence and error corrected the reads in the true UMI group, or by picking a random read from the UMI group in deduplication mode. For this study we used error corrected reads. (I) barplot of fraction of reads (y-axis) and their relative position on a transcript (x-axis) from the start or the end of the molecule depending on the gene length. (J) Scatter plot of short read (y-axis) versus long read (x-axis) gene expression depending on the length of the gene. (K) Scatter plot of ERCC concentration (y-axis) and ERCC molecules (x-axis) of mouse blastomere CELLO-seq data. (L) Scatter plot of ERCC concentration (y-axis) and ERCC molecules (x-axis) from mouse blastomere CELLO-seq libraries with Illumina sequencing. For J-L Pearson Correlation coefficient (R) and two-sided p-value shown.

**Extended Data Fig. 2:**
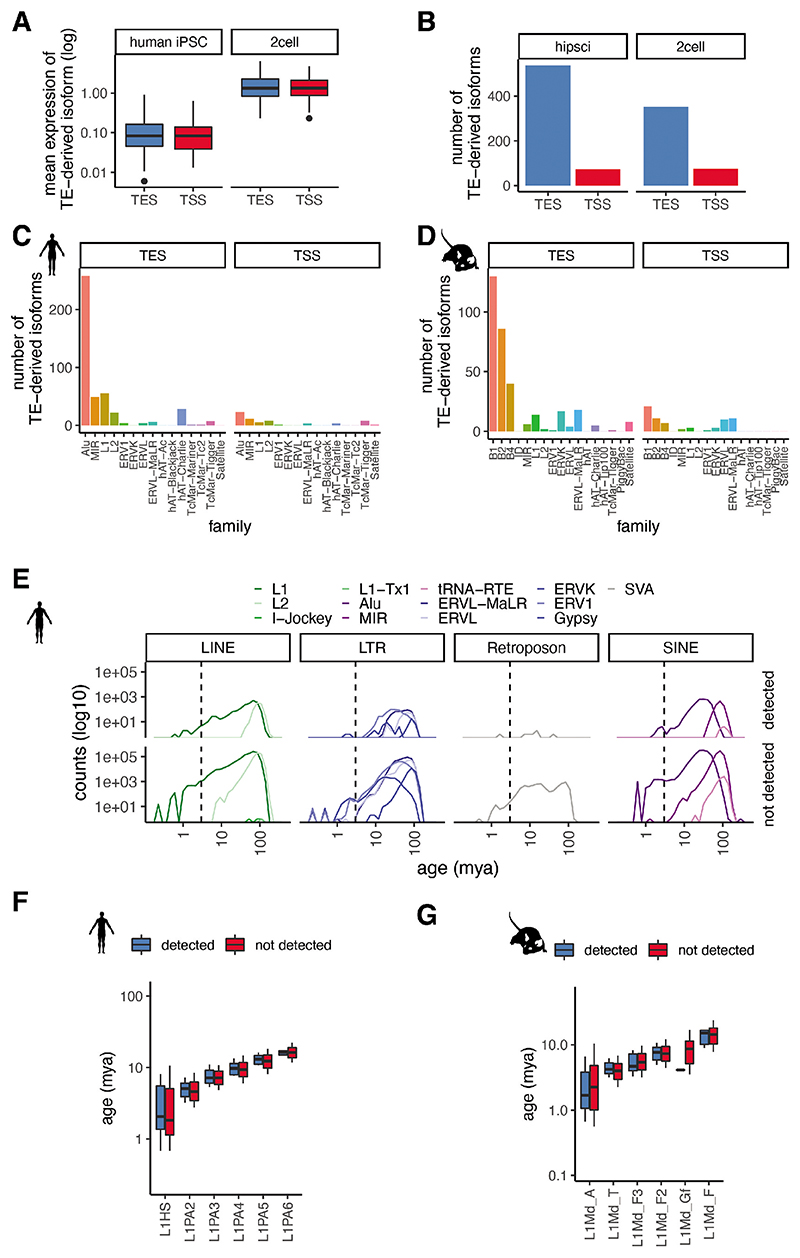
Isoform analysis. (A) expression of TE derived-isoforms in human iPSCs and mouse 2cell data stratified by whether a repeat acts as a transcript end site (TES) or as a transcript start site (TSS). Mouse: TES (n=353), TSS (n=76). Human: TES (n=537), TSS (n=73). (B) number of TE derived-isoforms in hiPSCs and mouse blastomeres with repeat as TES or TSS. (C) Barplot of repeat family underlying repeat derived-isoforms in hiPSCs. (D) Barplot of repeat family underlying repeat derived-isoforms in mouse blastomeres. (E) frequency plot of number of repeats (x-axis) by age of TE (mya) (y-axis). TEs that mapped to hiPSCs (top), all repeats from mouse UCSC repeatmasker annotation (bottom). TEs are grouped by class and colour-coded by family. (F) boxplots of age (mya) of TEs (y-axis) of young human L1s either detected or not detected in hiPSC dataset. detected: L1HS (n=15), L1PA2 (n=34), L1PA3 (n=34), L1PA4 (n=20), L1PA5 (n=18), L1PA6 (n=17), not detected: L1HS (n=1692), L1PA2 (n=5148), L1PA3 (n=11194), L1PA4 (n=12471), L1PA5 (n=11735), L1PA6 (n=6195). (G) boxplots of age (mya) of TEs (y-axis) of young mouse L1s either detected or not detected in mouse blastomeres. detected: L1MdA (n=27), L1MdT (n=44), L1MdF3 (n=8), L1MdF2 (n=43), L1MdGf (n=1), L1MdF (n=5), not detected: L1MdA (n=16817), L1MdT (n=23644), L1MdF3 (n=16138), L1MdF2 (n=64855), L1MdGf (n=1079), L1MdF (n=4011). The boxplots in A,F and G show the median, first and third quartiles as a box, and the whiskers indicate the most extreme data point within 1.5 lengths of the box.

**Extended Data Fig. 3:**
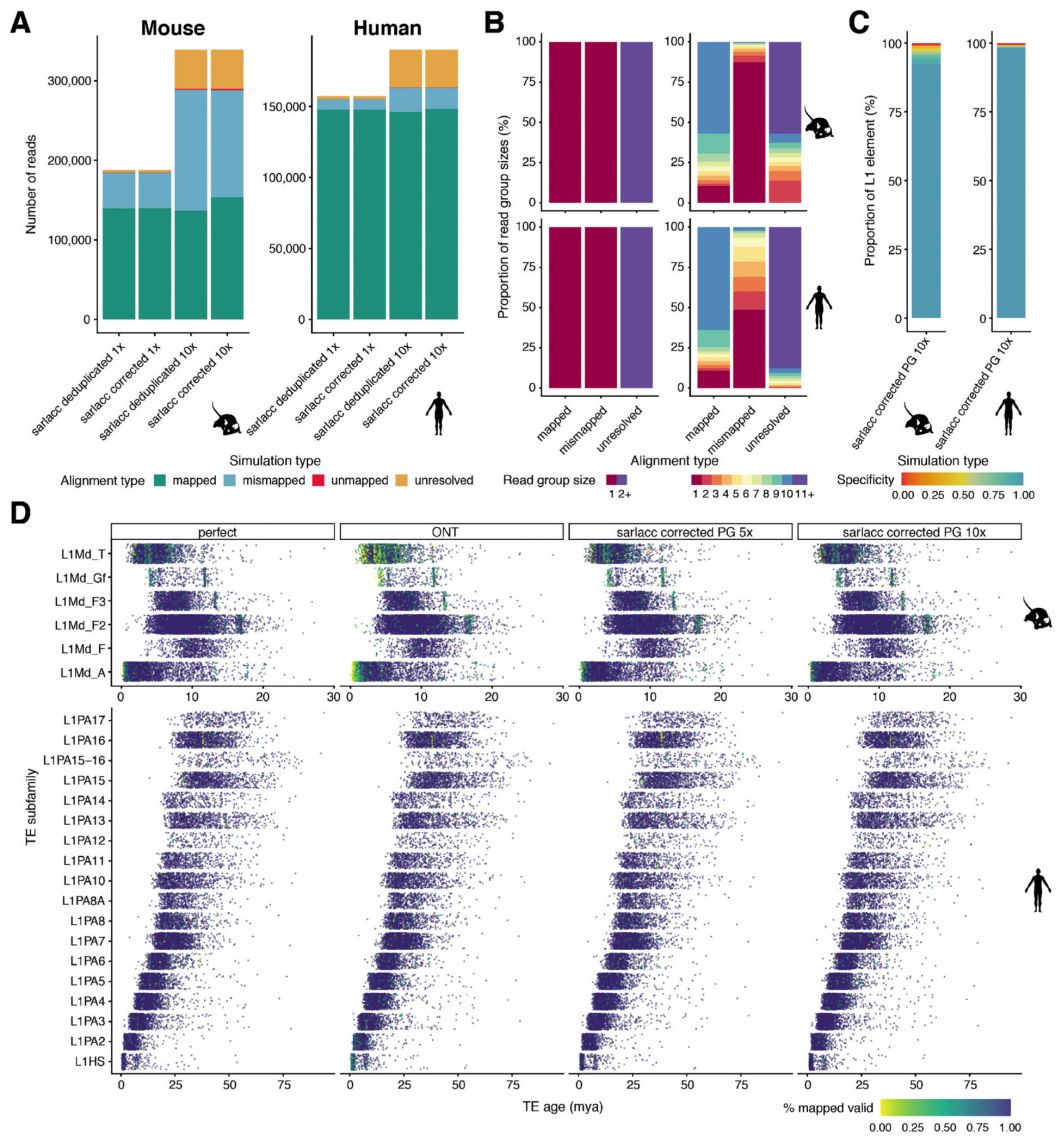
Simulation of correct mapping of young L1 in mouse and human genome. (A) bargraph showing the number of reads (y-axis) by the simulation type with either 1x or 10x coverage (x-axis), color-coded by alignment type with mapped = read at correct location after mapping with minimap2 to the genome, mismapped = read maps at wrong location, unmapped = read not mapped, unresolved = group has more than one molecule present and group cannot be resolved to a unique read. mouse (left), human (right). (B) bargraph of proportion of read group sizes (y-axis) by alignment type (x-axis), left showing 1x read coverage, right showing 10x read coverage. Color-coded by group size. mouse (top), human (bottom). (C) Stacked bargraph showing proportion of L1 elements (y-axis) by simulation type using 10x read coverage (x-axis), coloured by specificity score, mouse (left), human (right). (D) Jitter plot of TE subfamily (y-axis) by TE age (million years ago) grouped by simulation type and coloured by % of mapped reads with yellow being 0% mapped and dark blue being 100% mapped. Mouse L1 top panel and human L1 bottom panel. Simulation type: perfect = perfect read identity, ONT = ONT read identity, ONT 5x = ONT read identity with 5x coverage, sarlacc corrected 5x = ONT read identity score, 5x coverage with sarlacc error correction, sarlacc corrected 10x = ONT read identity score, 10x coverage with sarlacc error correction, sarlacc deduplicated 5x = ONT read identity score, 5x coverage with sarlacc deduplication by randomly choosing 1 read. PG = perfect grouping.

**Extended Data Fig. 4:**
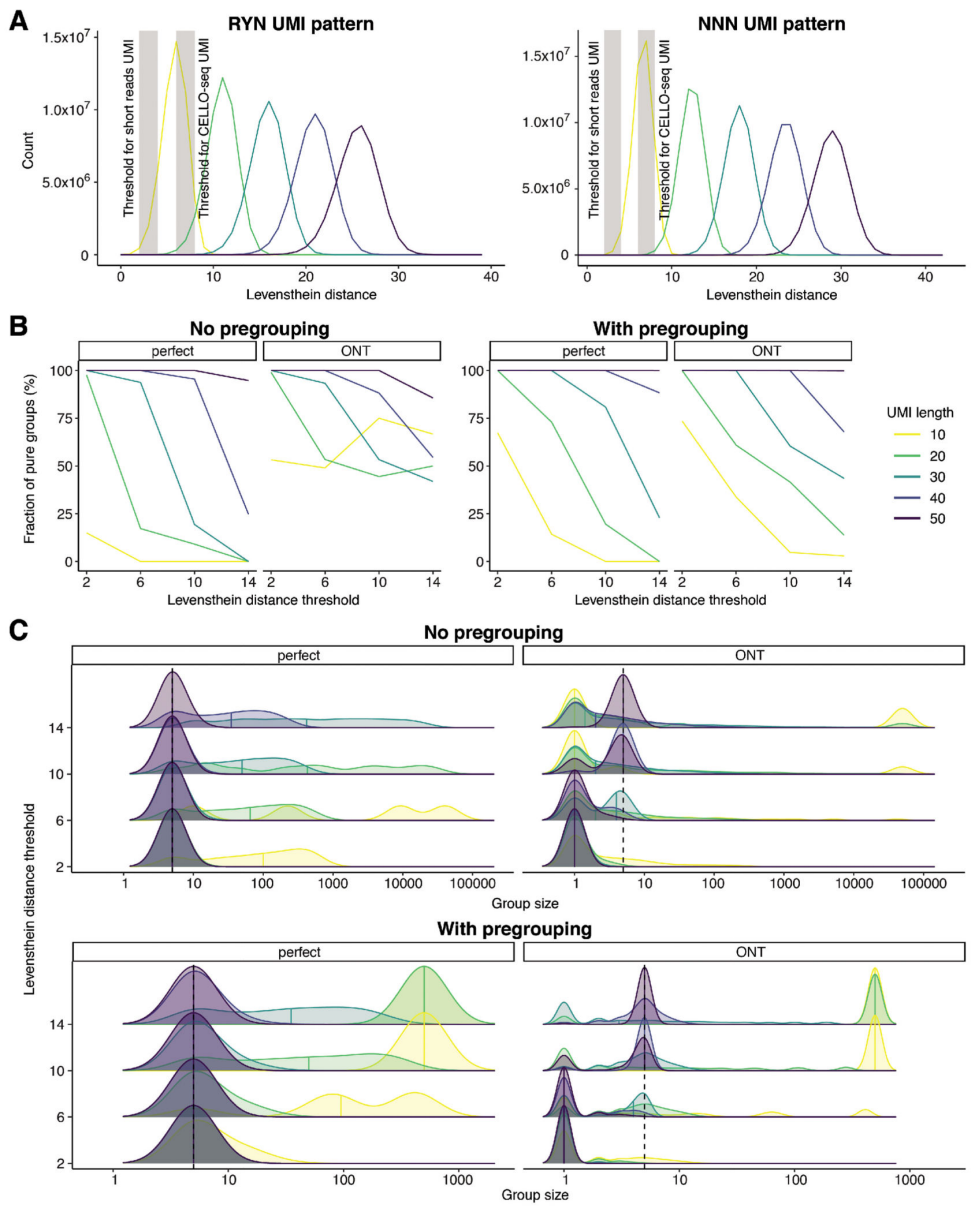
UMI simulations. (A) Distribution of Levenshtein distance between randomly simulated UMI (x-axis) based on UMI length with RYN pattern (left) or NNN pattern (right). Light grey bar shows distance threshold for grouping of reads by UMIs used for most short read UMIs or CELLO-seq. (B) Line graph of fraction of pure groups (y-axis) by levenshtein distance (x-axis) by UMI group, either with perfect read identity or ONT read identity. On the left is the line graph of UMI simulations without any pregrouping by mapping. On the right the line graph is UMI simulation where pregrouping was performed by random assignment of true UMI sequences into groups of 100 unique UMIs. (C) distribution plot of UMI group sizes (x-axis) by levenshtein distance threshold (y-axis) based on UMI length, with perfect of ONT read identity and no pregrouping (left) or pregrouping (right).

**Extended Data Fig. 5:**
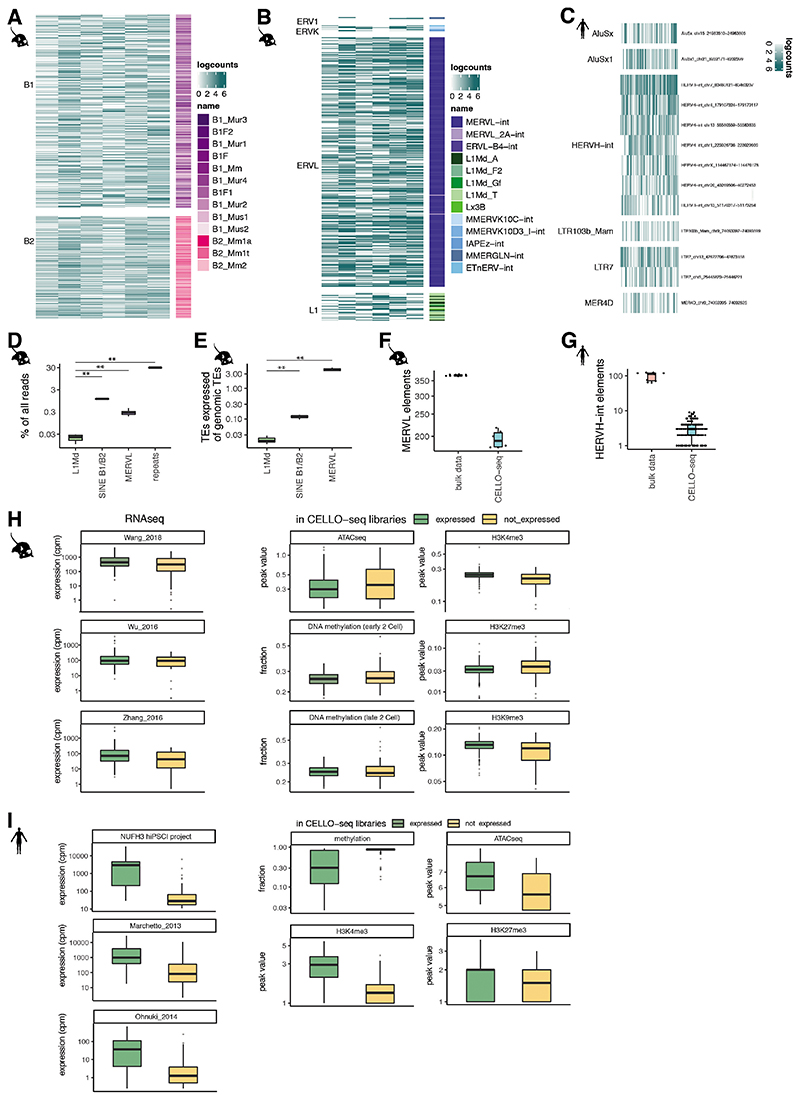
CELLO-seq to study locus specific TE expression. (A) Heatmap of expression of all SINE elements in mouse blastomeres, with rows clustered by SINE family and colour-coded by TE subfamily. (B) Heatmap of expression of full-length (>5000nt) elements in mouse blastomeres, with rows clustered by TE family and colour-coded by TE subfamily. (C) Heatmap of logcounts of highest expressed (mean expression > 1) elements in hiPSCs with rows clustered by TE subfamily. (D) Boxplot of percentage of reads mapped to TEs or TE families in CELLO-seq mouse 2-cells. P-value: L1Md to SINE B1/B2 =0.004998, L1Md to MERVL=0.004998, 2-sided Wilcoxon rank sum test. n=6 cells. (E) Boxplot of percentage of TEs expressed by number of TEs in the genome in CELLO-seq mouse blastomeres. p-value: repeats to L1Md=0.0022, repeats to SINE B1/B2= 0.0022, repeats to MERVL=0.0022, 2-sided Wilcoxon rank sum test. n=6 cells. (F) boxplot of number of MERVL elements expressed in each cell of CELLO-seq 2-cells compared to published short read data. CELLO-seq (n=6 cells), bulk (n=7 independent experiments). (G) boxplot of number of HERVH-int elements expressed in each cell of CELLO-seq compared to published short read data. CELLO-seq (n=96 cells), bulk (n=10 independent experiments). (H) expression, methylation, ATAC-seq and ChIP-seq of MERVL elements with read counts in CELLO-seq libraries compared to MERVL elements with no counts in CELLO-seq libraries. expressed (n=355 MERVLs), not expressed (n=41 MERVLs), datasets: ATAC-seq (n=1), DNA methylation (n=2), H3K27me3 (n=1), H3K4me3 (n=1), H3K9me3 (n=1), RNAseq (n=3). (I) expression, methylation, ATAC-seq and ChIP-seq data of HERV-int elements with read counts in CELLO-seq libraries compared to HERVH-int with no counts in CELLO-seq libraries. expressed (n=14 HERVH-ints), not expressed (n=110 HERVH-ints), each dataset (n=1), RNAseq (n=3). The boxplots shown in D-I show the median, first and third quartiles as a box, and the whiskers indicate the most extreme data point within 1.5 lengths of the box.

**Extended Data Fig. 6:**
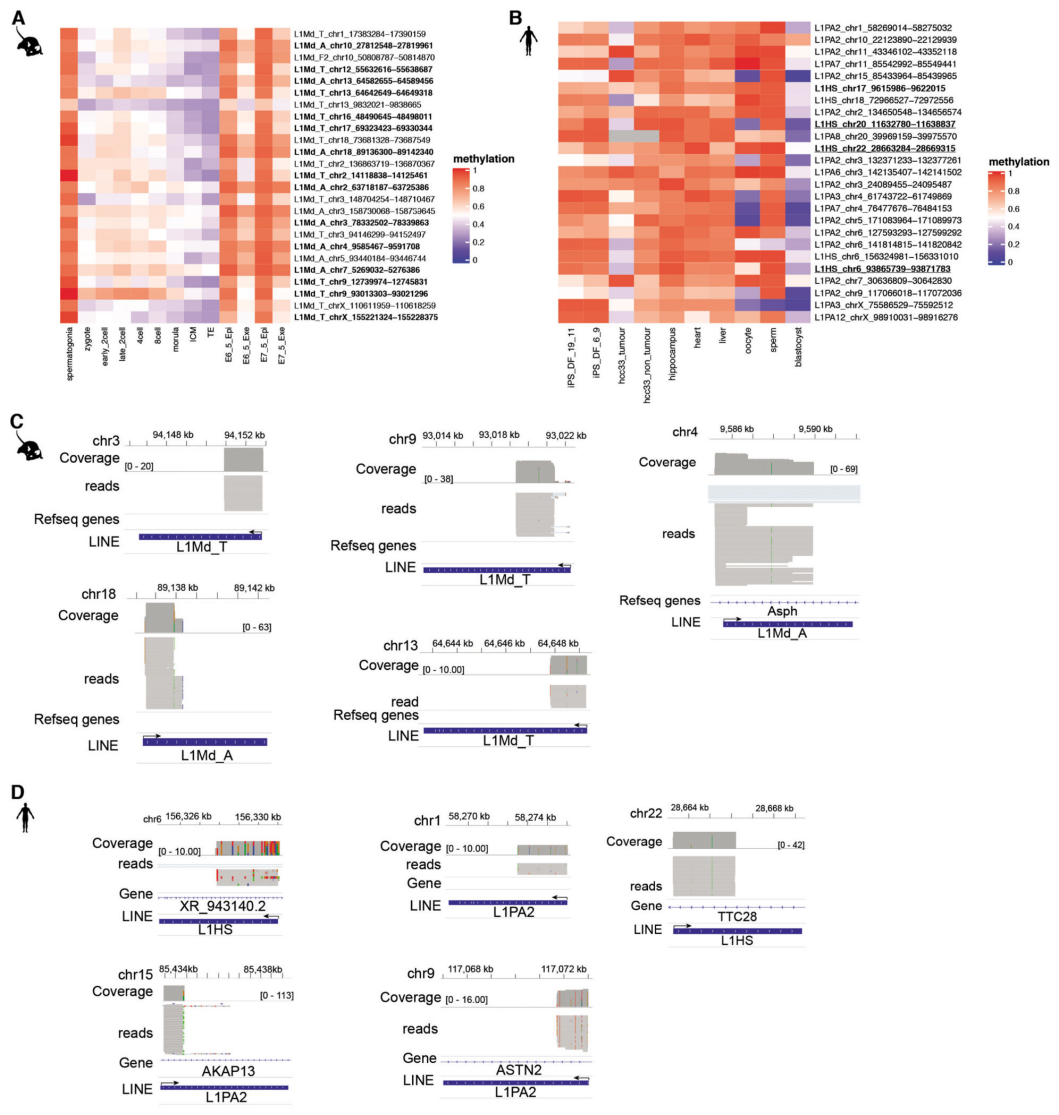
CELLO-seq to study locus specific young L1 expression. (A) DNA methylation of L1Md elements expressed in CELLO-seq mouse blastomeres. Methylation level of L1Mds across preimplantation development and in spermatogonia. Bold: L1s with full-length ORF by ORFfinder. (K) Methylation level of L1Mds across early development in human iPS cells as well as in tumour and normal tissue. Bold: L1s with full-length ORF by ORFfinder, underlined: L1s known to be mobile according to previous publications. (L) genome browser view of CELLO-seq reads overlapping young L1s (L) in mouse or (M) human. Arrows show direction of transcription of each L1 element.

## Supplementary Material

Supplementary Table 1

Supplementary Table 2

Supplementary Table 3

Supplementary Table 4

Supplementary Table 5

## Data Availability

The datasets generated during the current study are available under ArrayExpress accession E-MTAB-9577. We analysed 2cell RNAseq data from GSE97778, GSE66390, GSE76687, GSE71434; Atacseq from GSE76642, GSE66390; H3K9me3 data from GSE97778; H3K4me3 from GSE73952, GSE76687, GSE71434; H3K27me3 from GSE73952, GSE76687; and whole genome bisulfite data from GSE97778 and E-MTAB-9090. We analysed human iPSC RNAseq data from GSE47626, GSE56568; H3K4me3, H3K9me3, H3K27me3 and whole genome bisulfite data from GSE16265; and H3K4me3 from GSE16256.

## Abbreviations

CELLO-seq: CELl LOng read RNA sequencing
PacBio: Pacific Biosciences
MaLRs: Mammalian apparent LTR-retrotransposons
SNP: single nucleotide polymorphism
sc: single cell
TE: Transposable element
ORFs: Open reading frames
ASE: Allele specific expression
ERV: endogenous retrovirus
LINE: Long interspersed element
SINE: Short interspersed element
RNAseq: RNA sequencing
hiPSCs: Human induced pluripotent stem cells
RT: Reverse Transcription
LTRs: long terminal repeat elements
NGS: next generation sequencing
ONT: Oxford Nanopore technologies
UMIs: unique molecular identifiers
TSO: template switch oligo
ZNFs: Zinc finger nucleases
nt: Nucleotides
bp: Base pairs
TSS: Transcription start site
TES: Transcription end site
ERCC: External RNA Controls Consortium
